# Companies Committed to Responsible AI: From Principles towards Implementation and Regulation?

**DOI:** 10.1007/s13347-021-00474-3

**Published:** 2021-10-06

**Authors:** Paul B. de Laat

**Affiliations:** University of Groningen, Groningen, Netherlands

**Keywords:** Accountability, AI principles, Bias, Ethical code, Ethics washing, Explainability, Fairness, Privacy, Regulation, Responsible AI, Security, Standards

## Abstract

The term ‘responsible AI’ has been coined to denote AI that is fair and non-biased, transparent and explainable, secure and safe, privacy-proof, accountable, and to the benefit of mankind. Since 2016, a great many organizations have pledged allegiance to such principles. Amongst them are 24 AI companies that did so by posting a commitment of the kind on their website and/or by joining the ‘Partnership on AI’. By means of a comprehensive web search, two questions are addressed by this study: (1) Did the signatory companies actually try to implement these principles in practice, and if so, how? (2) What are their views on the role of other societal actors in steering AI towards the stated principles (the issue of regulation)? It is concluded that some three of the largest amongst them have carried out valuable steps towards implementation, in particular by developing and open sourcing new software tools. To them, charges of mere ‘ethics washing’ do not apply. Moreover, some 10 companies from both the USA and Europe have publicly endorsed the position that apart from self-regulation, AI is in urgent need of governmental regulation. They mostly advocate focussing regulation on high-risk applications of AI, a policy which to them represents the sensible middle course between laissez-faire on the one hand and outright bans on technologies on the other. The future shaping of standards, ethical codes, and laws as a result of these regulatory efforts remains, of course, to be determined.

## Introduction

Out of concern for the unprecedented pace of AI development and the ensuing social and moral problems from 2016 onwards, a great many organizations have issued statements of commitment to principles for AI. Companies, civil society organizations, single-issue groups, professional societies, academic organizations, and governmental institutions from mainly the Western world and Asia started to formulate statements of principle.

Scepticism sets in soon enough. Would organizations involved in AI really be making steps towards implementing these lofty principles in practice? After all, in comparison with medicine, several obstacles immediately catch the eye: the young AI community lacks common values, professional norms of good practice, tools to translate principles into practices, and mechanisms of accountability (Mittelstadt, 2019). A stronger backlash against the continuing flow of declarations of good intent by *for-profit companies* in particular articulated even sterner doubts: they are just trying to embellish their corporate image with superficial promises, and effective implementation of the principles in practice is bound to remain toothless. They are just in the business of ‘ethics washing’, a neologism coined by analogy with ‘green washing’ to denote the ‘self-interested adoption of appearances of ethical behaviour’ by technology companies (Bietti, 2020). The language of ethics is being instrumentalized for self-serving corporate ends. They hope that as a result, regulation proper can be weakened or kept at bay; ethics is thereby transformed into a novel form of industrial self-regulation (Wagner, 2018).^1^

So, we confront the following question: are the companies that have publicly committed themselves to AI principles actually trying to practice what they preach—albeit in the face of serious obstacles? Or are they effectively not trying, but just buying time from the ever-looming threat of increasing governmental regulation? Are they just engaging in a public relations offensive that signals their virtues while masking their lack of proper action? These questions of the number and value of actual efforts for implementing responsible AI and the intentions behind them are the main inspiration for this research.

The research zooms in on the firms’ attitudes to responsible AI from two angles. On the one hand, I ask myself whether the companies involved *did* try to implement AI principles in practice in their own companies and, if so, precisely how and to what extent. This includes efforts by these companies to act in concert with other companies and realize responsible AI amongst themselves (self-regulation). On the other hand, societal organizations of every kind—governmental ones included—are clamouring for more principled AI. Their declarations about responsible AI for the future vastly outnumber the declarations by the companies themselves about principled AI. To what extent are ‘committed’ companies willing to grant them a say in the AI issues involved? Phrased otherwise, what are their attitudes towards the issue of regulation proper? On the one hand, they may still—as is usually the case—consider regulation a catastrophic outcome to be avoided as supposedly stifling innovation and stress self-regulation as the preferred alternative. On the other hand, they may embrace regulation as a means to ward off social unrest about the new AI technologies unfolding. Which stance on AI regulation are they currently adopting?

At the outset, an important qualification has to be mentioned. Throughout, I have *only* selected principled companies that are substantially involved in AI practices: they have their own AI expertise, build their own algorithms and models, and as a rule have special departments or sections for AI/ML development. This enables them to advance the state of the art in AI/ML, to be innovators, not just followers. In particular, they have the capacity to pioneer fresh approaches to responsible AI. The outcomes of these AI efforts are used internally for their own products or processes, for selling AI (as software or cloud services) to clients, or for advising about AI—or, of course, for a combination of those activities. Such companies, to be referred to as ‘*AI companies*’, have exclusively been selected for further consideration since only those kinds of firms are able to change the character of AI and transform it into responsible AI in practice. Companies embracing AI principles that lack those AI capabilities can only be expected to promote the cause of responsible AI more modestly by focussing on such AI whenever they source AI solutions from elsewhere or by recommending responsible AI to their clients.

A caveat on method is also indicated here. In collecting these accounts of principles for and regulation of AI, I faced the problem that these often do not refer to AI in general but more narrowly to the particular AI that is embedded in a company’s business processes or product offerings. When Facebook talks about AI, it is from the perspective of the AI embedded in their platform services. When Philips or Health Catalyst talk about AI, they do so with their medical applications of AI in mind. When Google talks about AI, they have their much broader spectrum of AI applications in mind—from search engines, natural language understanding applications, self-driving cars, to drones. Vice versa, if we start from the angle of technology, views about, say, facial recognition technology are mainly expressed by companies that actually sell services of the kind (such as Amazon, Microsoft, and IBM). So, comments about AI principles or AI regulation are usually delivered from a specific ‘corner’ of AI, either narrow or broad, without being specified as such. It therefore remains imperative for readers—as well as this author—to always consider the corporate context and be on the alert for possible incompatibilities between accounts.

For a broad definition of the AI involved in these accounts, the reader may usefully be referred to a document from the ‘High-Level Expert Group on AI’ (AI HLEG) which describes the joint understanding of AI that the group uses in its work. This describes AI as a system composed of perception, reasoning/decision-making, and actuation; and as a discipline including machine learning, machine reasoning, and robotics.^2^

## AI Companies and AI Principles

This research began with a precise identification of the AI companies that have embraced principles for AI. Several projects across the globe compile inventories of organizations *in general* subscribing to AI guidelines/principles or issuing statements/studies about AI governance. The most important source was the article by Jobin et al. (2019) who performed a carefully tailored web search. Other important sources that I have consulted include the following (in alphabetical order of their URLs): the web log maintained by Alan Winfield,^3^ a Harvard study from the Berkman Klein Center,^4^ the Future of Life Institute,^5^ the 2019 AI Index Report,^6^ AlgorithmWatch,^7^ the University of Oxford website maintained by Paula Boddington,^8^ and the AI Governance Database maintained by NESTA.^9^

However, these lists lump together all kinds of organizations that subscribe to principles for AI. So, as a first task, *companies* had to be disentangled from these lists. Subsequently, duplicates amongst them were removed and only proper ‘AI companies’ retained. This yielded 18 results. For the sake of completeness and in order to catch the most recent developments, I performed a supplementary web search for AI companies subscribing to AI principles (for details on search method, see Appendix). This only yielded one more result (Philips).

Further, the Partnership on AI has been taken into consideration. This early coalition (2016) of large AI companies (Amazon, Apple, Facebook, Google, IBM, and Microsoft) focusses on the development of benchmarks and best practices for AI. At present, any organization is welcome in this multi-stakeholder organization, as long as they ‘submit an expression of interest, signed by its authorized representative declaring a commitment to: [e]ndeavor to uphold the Tenets of the Partnership and support the Partnership’s purposes’ and ‘[p]romote accountability with respect to implementation of the Tenets and of the best practices which the PAI community generates […]’.^10^ Therefore, for the purposes of this investigation, companies that became partners of the PAI can also be considered to be committed to AI principles. In total, I counted 17 corporate PAI members that were proper AI companies; five were new names, so they were added to my list (Amazon, Apple, Facebook, Health Catalyst, Affectiva).

Finally, the AI HLEG has been inspected. This temporary group was formed by the European Commission (mid-2018) to advise on the implementation of AI across Europe. It consists of 52 experts, who are mainly appointed in their personal capacity (17 so-called type A members) or as organization representatives (29 type C members). Several of those type C members come from firms: 12 in all.^11^ Should their participation be considered a commitment to AI principles and their companies candidates for my list? I would argue that participation in the AI HLEG falls short of such a commitment. For one thing, the selection criteria do not require adherence to any kind of principles—it is just expertise that counts.^12^ For another, as far as their final report is concerned, members only ‘support the overall framework for Trustworthy AI put forward in these Guidelines, although they do not necessarily agree with every single statement in the document’.^13^ In view of both considerations, participation in the AI HLEG cannot be interpreted as fully binding any firm—or any other organization for that matter—to principles for AI. The Expert Group is a political arena for developing a common standard for AI principles, not a forum for commitment to it.

As a result of this exercise, I obtained 24 ‘committed’ AI companies in total; these are listed in Table 1. Note that the whole search procedure was conducted in English, leaving out any committed AI companies that exclusively (or predominantly) publish their company documents in another language. Moreover, Chinese companies such as Tencent and Baidu have been left out, since apart from the hurdle of language, the Chinese political system is hardly to be compared with that of the USA or Europe; comparing statements about the ethics and governance of AI would be a strenuous exercise. Further, many companies with clear commitment to AI principles have been left out where my second criterion was not met: they do not have substantial AI capabilities of their own (such as *The New York Times*, Zalando), have just started their AI efforts (Telia), or went commercial only recently (OpenAI).^14^

**Table 1:** AI companies that committed to AI principles (ordered by revenue)^1^

	Type of industry Headquarters’ location	Commitment to AI principles	Member of ‘Partnership on AI’
Amazon	E-commerce, cloud computing Seattle, USA		+
Apple	Hardware and software, services Cupertino, USA		+
Samsung	Electronics, semiconductors Suwon, South Korea	*	+
Google	Internet, cloud computing, software Mountain View, USA	*	+
Microsoft	Hardware, software, electronics Redmond, USA	*	+
Deutsche Telekom	Telecommunications Bonn, Germany	*	+
Sony	Audio, video, photography Tokyo, Japan	*	+
IBM	Cloud computing, AI, hardware and software New York, USA	*	+
Intel Corporation	Semiconductors Santa Clara, USA	*	+
Facebook	Social media Seattle, USA		+
Telefónica	Telecommunications Madrid, Spain	*	
Accenture	Management consulting Dublin, Ireland	*	+
SAP	Enterprise software Walldorf, Germany	*	
Philips	Consumer electronics, healthcare Amsterdam, the Netherlands	*	
Salesforce	CRM services San Francisco, US	*	+
McKinsey (Quantum-Black)^2^	Management consulting (data analytics) (No headquarters)	*	+
Sage	Enterprise software Newcastle upon Tyne, UK	*	
TietoEVRY	Enterprise software Helsinki, Finland	*	
Kakao	Social media, services Jeju City, South Korea	*	
Unity Technologies	Video games San Francisco, USA	*	
Health Catalyst	Medical data analytics Salt Lake City, USA		+
DeepMind (Google)^3^	AI London, UK	*	+
Element AI	AI Montreal, Canada	*	+
Affectiva	Emotion AI Boston, USA		+

^1^Note that Deutsche Telekom, Salesforce, Health Catalyst, and Element AI originally were partners of the PAI and have therefore been listed as such. At the time of finishing this manuscript (early 2021), however, they appear to have cancelled their membership. Note also that PAI member OpenAI, a general purpose AI research laboratory, has been left out since it went commercial only very recently (their GTP-3 language model was launched in June 2020).

^2^Quantum-Black is the data analytics arm of McKinsey.

^3^As a research laboratory, DeepMind enjoys considerable independence within Google. They also issued AI principles of their own (long before Google did so). Therefore, DeepMind is listed separately from Google.

After having identified these 24 ‘committed’ AI companies, I proceeded to delve deeper into their commitments. What exactly are the component parts of their declarations of principle? This exercise is not unimportant; after all, subsequently, I take these firms at their word and investigate whether they practice what they preach. I went back to the statements about AI principles for each of these companies (as well as the PAI) looking for the precise way in which the AI principles or guidelines were publicly formulated. The results are listed in Table 2.

**Table 2:** Exact phrasing of AI principles by the PAI and the 19 AI companies with explicit commitment to AI principles

PAI and AI companies committed to AI principles	Their own phrasing of ‘AI principles’	Fairness, justice	Transparency, explainability	Security, safety	Privacy	Responsibility, accountability	Broader principles
Partnership on AI	Thematic pillars; tenets	No bias, fairness, inclusivity	Transparency, explainability, interpretability, understandability	Safety, security, reliability, robustness	Privacy	Accountability	Trustworthiness; empowering people; study the social and economic impact of AI; no AI that violates international conventions or human rights; open dialogue and engagement with stakeholders
Samsung	Principles for AI ethics	No unfair bias, equal access, equality and diversity	Transparency, explainability	Security		Social and ethical responsibility	To the benefit of society; for corporate citizenship
Google	AI principles	No unfair bias	Relevant explanations	Safety, security	Privacy	Accountability	Humans in control of AI; uphold scientific excellence; AI to be socially beneficial; no pursuit of AI that causes overall harm or injures people, is intended for illegitimate surveillance purposes, or contravenes international law/human rights
Microsoft	Principles for responsible AI	Fairness, inclusiveness	Transparency, intelligibility	Safety, reliability	Data privacy and security	Accountability	
Deutsche Telekom	AI guidelines	No biased data, fairness	Transparency, no black boxes	Data security, robustness	Data privacy	Auditability, responsibility	Trust; supporting customers; cooperation between human and machine; engagement with stakeholders; sharing our know-how
Sony	AI ethics guidelines	Fairness, diversity	Transparency, explainability	Safety, security	Privacy		Respect for human rights; support for creative lifestyles and a better society; engagement with stakeholders; fostering AI human talent
IBM	Trust and transparency principles for AI	No bias, no discrimination, fairness	Transparency, explainability	Data security, robustness	Data privacy	Accountability	Client control of data; value alignment; augment human intelligence
Intel*	None	No biased data, no discrimination	Explainability	Security (from cyber-attacks)	Privacy	Accountability	
Telefónica	AI principles	No bias, no discrimination, fairness	Transparency, explainability	Security	Privacy		Checking the veracity of data and logic used by suppliers; benefits for people; humans in control of AI; respect for human rights and sustainable development
Accenture*	Responsible AI	No bias, no discrimination, equality, diversity	Transparency, explainability	Security	Data protection, Privacy	Liability	Human values
SAP	Guiding principles for AI	No bias, no discrimination, inclusivity	Transparency	Safety, security, reliability	Data protection, privacy		Respect for human rights; empowering people; engaging with wider society to discuss economic and social impact and normative issues
Philips	AI principles, data principles	No bias, no discrimination, fairness	Transparency	Security (as data principle), robustness against misuse	Privacy (as data principle)		Human supervision of AI; people’s well-being; medical care for all; sustainable development
Salesforce	AI ethics	Inclusivity	Transparency, explainability	Safety		Accountability	Customer control of data and models; empowering and benefitting society; respect for human rights
McKinsey (Quantum Black)*	Responsible AI	No bias, fairness	Explainability		Privacy		
Sage	Core principles for ethical and responsible AI	No bias, inclusivity		Safety, security		Accountability	Align with human values; cooperation between AI and humans
Tieto	AI ethics guidelines	No bias, fairness, inclusivity	Transparency, explainability	Safety, security		Responsibility	AI for good; respect for human rights
Kakao	Algorithm ethics	No bias	Explainability	Security			Ethical data collection and management; enhance benefit and well-being of mankind; embrace our society
Unity Technologies	Guiding principles for ethical AI	No bias	Transparency		Data protection	Accountability, responsibility	No manipulation of users; respect for human rights
Deep Mind (Google subsidiary)*	Responsible AI	No bias, no discrimination, fairness, inclusivity	Transparency		Privacy	Accountability	Beware of misuse and unintended consequences; consider social and economic impact
Element AI	Responsible AI	Fairness	Transparency, explainability	Safety		Accountability	Individual control of personal data; humans in control of AI; respect for human rights; no offensive weapons for the military/police; sustainable development
Number of firms mentioning:	n/a	19	18	17	13	13	16

^*^Dispersed over various documents

Notes: Companies ordered by revenue. The 5 companies without explicit commitment to AI principles (members only of the PAI) are not listed in the table since they only conform to the principles of the PAI. The principles are displayed in the order of their empirical frequencies amongst the 19 companies (see last row). The headings for the 6 columns are mine. Accuracy (mentioned by Telefónica only) deleted since part of normal requirements. Safety and privacy partly overlap for ML; therefore, they are considered together. Adjectives from the original sources in columns 3–7 converted to nouns (transparent → transparency, etc.) for easier comparison. Some companies in addition drafted more general codes of conduct; these codes are not considered here. Links to the documents that I drew my information from are available on request.

In the first row, the tenets of the PAI are explicated. In the rows below, results are tabulated for the 19 companies with explicit declarations of AI principles (leaving out the five members-only of the PAI). Throughout, I classified the terms in use under six headings, ordered according to the empirical frequencies obtained.^15^ Table 2 shows clearly that almost all firms emphasize four to five of the five main principles for AI: fairness/justice, transparency/explainability, security/safety, privacy, and responsibility/accountability. Only three firms (out of 19 in all) mention just three of them. So, a clear and homogeneous sense of purpose emerges as far as responsible AI is concerned: AI is to be fair and just, transparent and explainable, secure and safe, and privacy-proof, with responsibility and accountability taken care of; broader principles such as humans in control, benefitting society, respect for human rights, and not causing harm are stressed in various combinations. Note that this conception—not coincidentally of course—matches closely the term ‘trustworthy AI’ that has been coined in EU circles, the AI HLEG in particular. Five of their seven ‘key requirements’ for trustworthy AI (which is, in addition to being lawful, both ‘ethical’ and ‘robust’) match one to one with the above; their requirements of ‘human agency and oversight’ and ‘societal and environmental well-being’ correspond to my ‘broader principles’.^16^

Below, I zoom in on these five ‘core’ principles, as representing the common denominator of the promise made by the 24 firms committed to AI. Notice that the first four of these principles imply requirements on the *technical core* of AI: the methods of ML have to change for them to be satisfied. Without this transformation, these principles cannot be satisfied—let alone any of the other more general principles associated with responsible AI (the ‘broader principles’ in Table 2).

A caveat is in order: the homogeneity may look impressive, but the terms employed leave ample room for interpretation. While, for example, security and robustness have acquired quite circumscribed meanings, transparency and explainability are more ambiguous. Do they refer to clarifying how predictions were produced? To the importance of features involved in a specific prediction? To counterfactual-like clarifications? So, all depends on how the committed companies are actually going about these challenges in practice.

## Implementation of AI Principles Inside the Firm

Very little literature is available about corporate implementation of AI principles. While Darrell West is one the first authors to write about the issue,^17^ Ronald Sandler and John Basl, in an Accenture report, paint the following broader picture based on several additional sources.^18^ Upon acceptance of an AI code of ethics, an ethics advisory group (or council) is to be installed at the top of an organization in order to import outside viewpoints and expertise, and an ethical committee (or review board), led by a chief data/AI officer, is to be created which provides guidance on AI policy and evaluates AI projects in progress.^19^ In both groups, ethicists, social scientists, and lawyers are to be represented. Further, auditing as well as risk and liability assessments have to become standard procedures for AI product lines and products. Keeping track of audit trails is to support the auditing. Finally, training programmes for ethical AI are to be implemented, and a means for remediation provided in case AI inflicts damage or causes harm to consumers. Note that West reported that in a US public survey, many of these ‘ethical safeguards’ obtained the support of 60–70% of the respondents.^20^

A further source is a report produced by several governmental organizations from Singapore,^21^ which presents a state-of-the-art manual for implementing responsible AI within the existing governance structures of organizations in general (p. 16). Particularly interesting for my purposes are their proposals (p. 21 ff.) for the introduction of adequate governance structures (such as an AI review board), clear roles and responsibilities concerning ethical AI (e.g., for risk management and risk control of the algorithmic production process), and staff training. Further, they propose ways to put ‘operations management’ (i.e., management of the processes of handling data and producing models from them by means of ML) on a more ethical trajectory (p. 35 ff.). They suggest an exhaustive series of requirements: accountability for data practices (including attention to possible bias) (p. 36 ff.), explainability, repeatability, robustness, traceability, reproducibility, and auditability (p. 43 ff.).

An aspect that is mostly glossed over in these sources is that requirements like the absence of bias, explainability, and robustness in particular cannot adequately be met by citing measures culled from management handbooks alone—especially as new techniques of ML will have to be invented and associated software tools be coded from scratch.

With this broad canvas in mind, to what extent did the 24 committed companies implement their AI principles in corporate practice? I performed a web search to discover associated mechanisms of implementation of AI principles (see Appendix for the method, searching with KS2). Which implementations did the committed companies report of their own accord? All results are rendered in succinct form in Table 3.

At the outset, it is to be reported that seven of my committed firms did not publish anything about these issues (or, for that matter, about their attitudes towards (self-)regulation of AI—on which I report later). So, there is simply no material to be inserted under the headings of Table 3—the entries for them have remained empty throughout my search efforts (with KS2, see Appendix). Inside some of these firms, I did trace discussions about AI being conducted, but clear results have not been published. This specifically concerns Apple, Samsung, Deutsche Telekom, Sony, Kakao, Unity Technologies, and Affectiva. Therefore, in the sequel, I no longer take them into consideration; specifically, their names do not appear in Table 3.

**Table 3:** Companies practicing and developing AI in order to apply/sell/advise about AI that explicitly have committed to principles or guidelines for AI and/or to the tenets for AI of the Partnership on AI—tabulated according to their internal governance for responsible AI; training and educational materials about responsible AI; new tools for fair/non-biased AI and explainable AI and secure/privacy-proof AI and accountability of AI; and their external collaboration and funding for research into responsible AI

AI companies committed to AI principles^1^	Governance for responsible AI inside the firm^2^	Training and educational materials produced about responsible AI	New tools for fair/non-biased AI	New tools for explainable AI	New tools for secure/privacy-proof AI^3^	New tools for accountability of AI	External collaboration and funding for responsible AI research
Amazon				SHAP values and feature importance tools (proprietary)			Co-funding of NSF project ‘Fairness in AI’
Google	Advanced Technology External Advisory Council (now defunct); Ethics & Society team; responsible innovation teams review new projects for conformity to AI principles	Employee training about ethical AI, educational materials (see ‘People + AI Guidebook’)	Facets, What-If tool, Fairness Indicators (all open source)	What-If tool (open source)	CleverHans (open source); Private Aggregation of Teacher Ensembles (open source), Tensor Flow Privacy (open source); Federated Learning, RAPPOR, Cobalt (open source)	Model cards	
Microsoft	AI and Ethics in Engineering Research Committee, Office of Responsible AI	Internal guidelines and checklists (e.g., ‘In Pursuit of Inclusive AI’, ‘Inclusive Design’)	FairLearn (open source)	InterpretML (open source)	WhiteNoise package (open source)	Data sheets for datasets	
IBM	AI Ethics Board (chaired by AI Ethics Global Leader and Chief Privacy Officer)	Guidelines for AI developers (‘Everyday Ethics for AI’)	AI Fairness 360 Toolkit (open source)	AI Explainability 360 Toolkit (open source)	Adversarial Robustness 360 Toolbox (open source)	Fact sheets	Joint research with Institute for Human-Centred AI (Stanford University), funding of Tech Ethics Lab (University of Notre Dame)
Intel	AI Ethics and Human Rights Team						
Facebook	AI Ethics Team		Fairness Flow (proprietary)	Captum (for deep neural networks) (open source)			Funding of Institute of Ethics in AI (TU Munich)
Telefónica		AI Ethics course and AI Ethics self-assessment (for employees)					
Accenture		Advocates ‘responsible, explainable, citizen AI’ to clients, educational materials	AI Fairness Tool, part of AI Launchpad (proprietary)				
SAP	AI Ethics Advisory Panel, AI Ethics Steering Committee; diverse and interdisciplinary teams	Course about ‘trustworthy AI’ (for employees and other stakeholders)					
Philips							
Salesforce	Ethical Use Advisory Council, Office of Ethical and Humane Use of Technology, data science review board; inclusive teams	Teaching module about bias in AI (for employees and clients)	Einstein discovery tools (proprietary)	Einstein discovery tools (proprietary)			
McKinsey (Quantum Black)		Advocates ‘responsible AI’ approach to clients, educational materials		CausalNex (open source)			
Sage	Team diversity						
Tieto		In-company ethics certification, special AI ethics engineers, and trainers appointed					
Health Catalyst							
Deep Mind (Google subsidiary)	External ‘fellows’, Ethics Board, Ethics and Society Team						
Element AI	Team diversity	Internal blogposts about responsible AI	Fairness tools (proprietary)	Explainability tools (proprietary)			

^1^Companies are ordered by revenue. Apple, Samsung, Deutsche Telekom, Sony, Kakao, Unity Technologies, and Affectiva have been omitted from the table since my searches yielded no results for them.

^2^ ‘Ethics Team’ denotes what is variously referred to as ethical/ethics/review committee/board (highest corporate body concerning affairs of ethical AI).

^3^Techniques such as privacy by design (de-identification of data) and security by design (encryption) are not mentioned in the table since they are well-known and do not refer specifically to problems of ML.

Note: The sources that I drew my information in the table from are for the most part given in footnotes in the text of the article; otherwise, the sources are available on request.

### Internal Governance for Responsible AI

As far as governance for responsible AI is concerned, implementations varied from substantial to marginal to none at all (for this section as a whole: cf. Table 3). More than half of the committed firms had not introduced any concrete steps towards responsible AI (14 out of 24 companies).^22^ To be sure, they had discussions about it internally, yet nothing materialized that became public. Two others have at least taken steps to diversify the composition of their teams, as a contribution to the reduction of bias in AI (Sage, Element AI). The eight remaining companies did indeed introduce governance mechanisms at the top as suggested by West, Sandler and Basl, and the Singapore manual: an ethics advisory group (or council) for external input, and/or an ethical committee (or review board) that installs and oversees teams and/or working groups. However, the setup in full is only adopted by the smaller firms amongst them: SAP, Salesforce,^23^ and DeepMind.^24^ With the five remaining larger firms on my list (Google, Microsoft, IBM, Intel, and Facebook), input from the outside world did not materialize: an ethics advisory group has simply not been installed—only an ethical committee or review board.^25^ The firms in question will surely argue that they have ample contacts and consultations with ‘outsiders’. This is undoubtedly true, but the noncommittal nature of these interactions does not signal much of a willingness to grant influence to other societal actors.

Let me illustrate the workings of such internal governance with two examples. SAP’s ‘AI ethics steering committee’ is composed of senior leaders from across the organization. After having formulated SAP’s ‘guiding principles for AI’, it now ‘focuses on SAP’s internal processes and on the products that result from them, ensuring that the software is built in line with ethical principles’. At the same time, they installed an external ‘AI ethics advisory panel’, with ‘experts from academia, politics, and business whose specialisms are at the interface between ethics and technology—AI in particular’.^26^ Their setup is transparent— the names of all the panel members are made public.^27^

The ethical committee that Microsoft has installed is called AETHER (AI Ethics in Engineering and Research). Composed of ‘experts in key areas of responsible AI, engineering leadership, and representatives nominated by leaders from major divisions’,^28^ it gives advice and develops recommendations about AI innovation across all Microsoft divisions. In particular, it has installed working groups that focus on subthemes like ‘AI Bias and Fairness’ and ‘AI Intelligibility and Explanation’. This AETHER Committee uses the services of the Office of Responsible AI that convenes teams to ensure that products align with AI principles. Moreover, the Office takes care of public policy formulation and the review of ‘sensitive use cases’ (such as facial recognition software) related to responsible AI.

### The New Practice of Responsible AI: Training, Techniques, and Tools

Let us next turn to ‘operations management’ and the measures taken to transform it along the requirements of responsible AI. Companies committed to responsible AI have to ask new questions and develop new insights. The problem that they face is that most of the principles involved—fairness, explainability, security, and privacy—strike at the very heart of ML: the ways in which to perform ML have to be reflected upon. For these complex issues, proper research into the fundamentals of ML has to be performed. But research alone is not enough. ML and AI are eminently practical exercises, looking for the best algorithmic ways to produce models that can be used in practice. Therefore, at the end of the day, fresh research insights have to be translated into fresh software tools that update current practices. These tools allow taking ethical questions into account and may help in deciding about the most ethical course of action.

Against this background, I have been looking for new materials (blog posts, guidelines, manuals, checklists, techniques, tools, and the like) that have been developed by the 24 committed companies—with a particular focus on new software techniques and software tools. The results are reported in Table 3.

Half of all the 24 companies on my list, again, did not produce materials of any kind or set up courses for their employees. The principles for responsible AI are simply not (yet) addressed at the level of their workforce. The other half *did* address the espoused principles, to varying degrees. Four of them introduced (mostly informal) trajectories for training for responsible AI, accompanied by appropriate materials, either inside the firm (Telefónica, SAP, and Tieto) or geared towards external clients (McKinsey).^29^ Another three were able to develop some software tools geared towards responsible AI and incorporate them into their existing products (Amazon, Facebook,^30^ and Element AI^31^). Consider Amazon, for example, one of the largest providers of software as a service. Their clients can build ML models with Amazon SageMaker; it now incorporates some (proprietary) explainability tools (delivering feature importance and SHAP values—more about these indicators below).

The remaining five firms have actively been developing *both* new materials to be used in training sessions (for their personnel and/or clients) and new software tools. The two smaller firms amongst them, Accenture and Salesforce, have developed some first steps. Let me delve deeper into the case of Salesforce, a company that sells CRM (customer relationship management) software. Their course is a module about bias in AI that they have put up on their online platform called Trailhead (freely accessible to everyone).^32^ Apart from mentioning the virtues of diversity, respect for human rights and the GDPR, the ‘crash course’ focusses on bias and fairness in their various forms. This leads to ethical questions about AI which the various employee ranks have to ask themselves. Ultimately, AI may just amplify those biases—so how to remove them from one’s datasets? The module suggests conducting ‘pre-mortems’ addressing issues of bias, performing a thorough technical analysis of one’s datasets, and remaining critical of one’s model both while it is being deployed and afterwards.

In addition to this module, they developed a few software tools for responsible AI. Clients can bring their own datasets to the Salesforce platform and develop ML models from them (‘Einstein Discovery Services’). These services contain new tools that act as ‘ethical guardrails’.^33^ Top predictive factors for a global model are produced upon request (→ explainability). Moreover, customers may define ‘protected fields’ (such as race, religion); that is, they are to receive equal treatment. The system then issues a warning whenever a proxy for them is detected in the dataset submitted for training (→ bias).

Training materials and software tools from Salesforce, though, are just tiny steps forward if we compare them with the offerings of the remaining three companies on my list: Google, Microsoft, and IBM. These easily outpace all the efforts mentioned so far in both quantity and quality. Their employee training has attained large proportions, and the materials for them stack up to an impressive list of notes, guides, manuals, and the like that probe deeply into the aspects of bias, fairness, explainability, security, privacy, and accountability.

Additionally, many of the new techniques involved have been encoded into programs in order to become effective. These are usually published on GitHub, as open source: other developers may download the source code and use or modify it. This move is not mere altruism, of course. The code involved may get better because of modifications contributed in return; the code may become a de facto standard; and it may help the company to attract clients to their commercial products. Notably, these new techniques and tools are largely the result of big research efforts by Google, Microsoft, and IBM into the features of bias, explainability, and security/privacy of ML. Since about 2016 onwards, their publication output in computer science journals, with a focus on these areas, has risen considerably.

Since the instruction materials from these three firms (perusable on their respective websites) are comparable to the Salesforce example above, below I focus on their software contributions. These are discussed in the order in which the features of responsible AI have become the subject of research: the issue of bias has been researched for a longer time, while the issues of explainability and security/privacy (insofar as arising from adversarial attacks) have come to the fore just recently.

#### Google, Microsoft, IBM: Fairness Tools

**Fig. 1:**
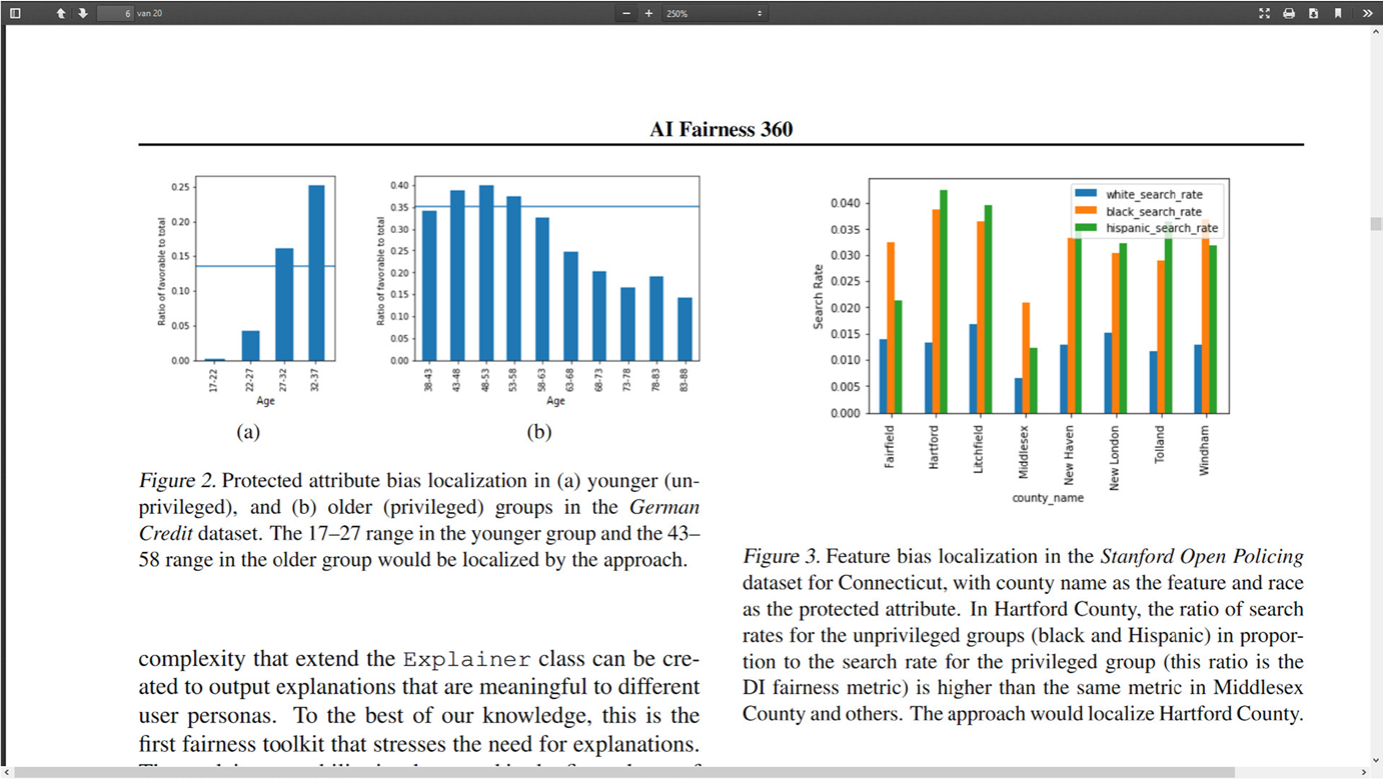
Localization of bias in datasets about creditworthiness (left) and police search rate (right) across privileged and unprivileged groups (IBM). Source: https://arxiv.org/pdf/1810.01943.pdf

**Fig. 2:**
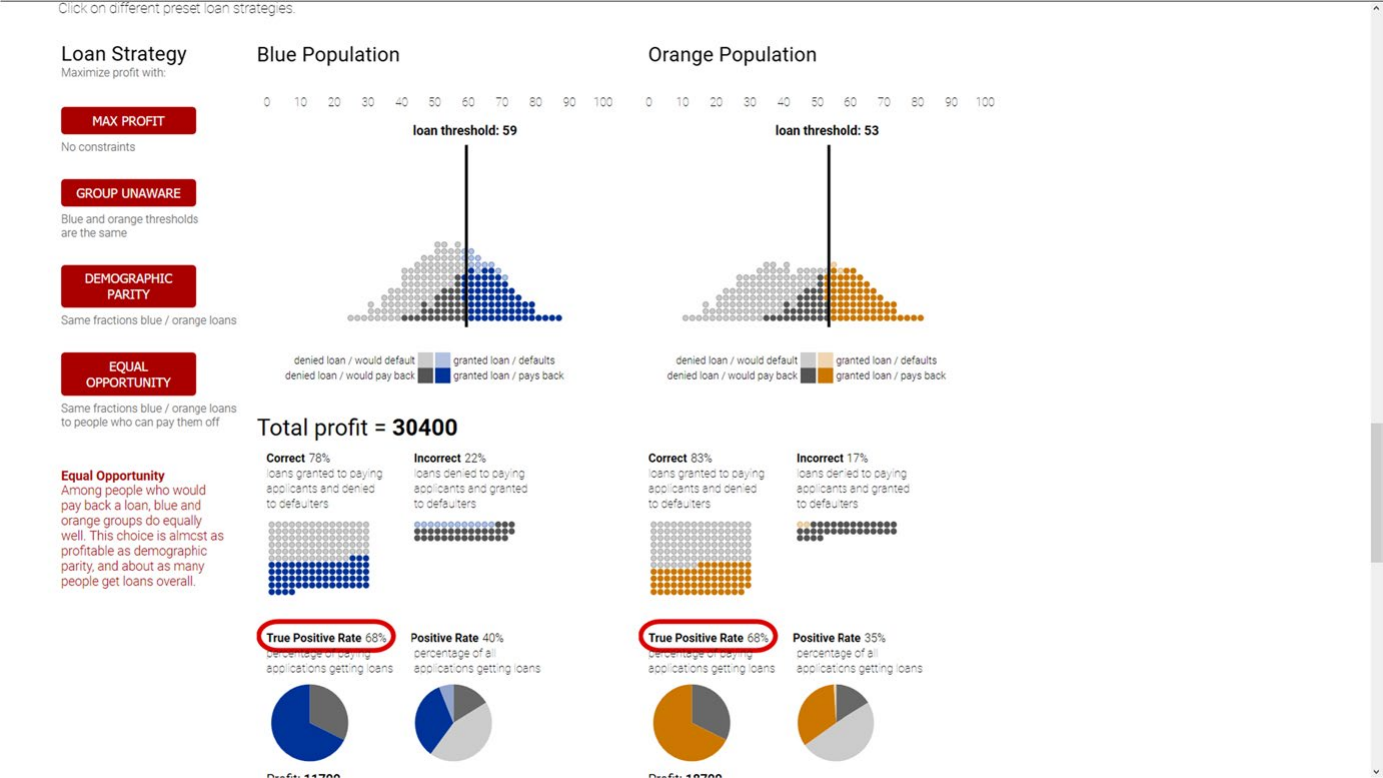
Various fairness measures and their thresholds: screenshot for equal opportunity (synthetic data from Google). Source: https://research.google.com/bigpicture/attacking-discrimination-in-ml/

The fairness issue derives from the observation that bias against one group or another may easily creep into the ML process: bias in datasets (cf. Fig. 1) translates into ML producing a biased model as output. As a consequence, the generated predictions discriminate against specific groups. In order to address this fairness issue, Google, Microsoft, and IBM have developed several techniques and tools (cf. Table 3). Let me go into Google’s offerings first. Facets Overview enables visualizing datasets intended to be used (e.g., allowing to detect groups that are not well represented, potentially leading to biased results).^34^ With the What-If tool, one may investigate the performance of learned models (classifiers, multi-class models, regression models) using appropriate test sets.^35^ It may, in particular, address fairness concerns by analysing that performance across protected groups. With the push of a button, several definitions of fairness (‘fairness indicators’) can be implemented for any such group: the threshold levels are shifted accordingly (cf. Fig. 2).^36^ Microsoft, on its part, offers similar analyses and tools bundled together in its Python package called FairLearn.^37^ It also delves into fairness metrics for the various relevant groups and measures to mitigate corresponding fairness concerns in the ML process.

In comparison, though, both ‘fairness packages’ are less developed than the one from IBM.^38^ Big Blue offers a larger menu of fairness measures which users may choose from (depending on the particular use case). For protected variables, metrics such as equal parity, demographic parity, and equal opportunity may be chosen. In order to mitigate biases, a range of techniques are presented—as invented by researchers from academia and industry, often working together. In general, training data may be processed before training starts (e.g., reweighing training data), the modelling itself can be adjusted (e.g., taking prejudices into account while processing the data), or biases in the algorithmic outcomes may be mitigated (e.g., by changing the model’s predictions in order to achieve more fairness). For most of the techniques involved (about 10 in all), IBM has developed software implementations.

#### Google, Microsoft, IBM: Explainability Tools

**Fig. 3:**
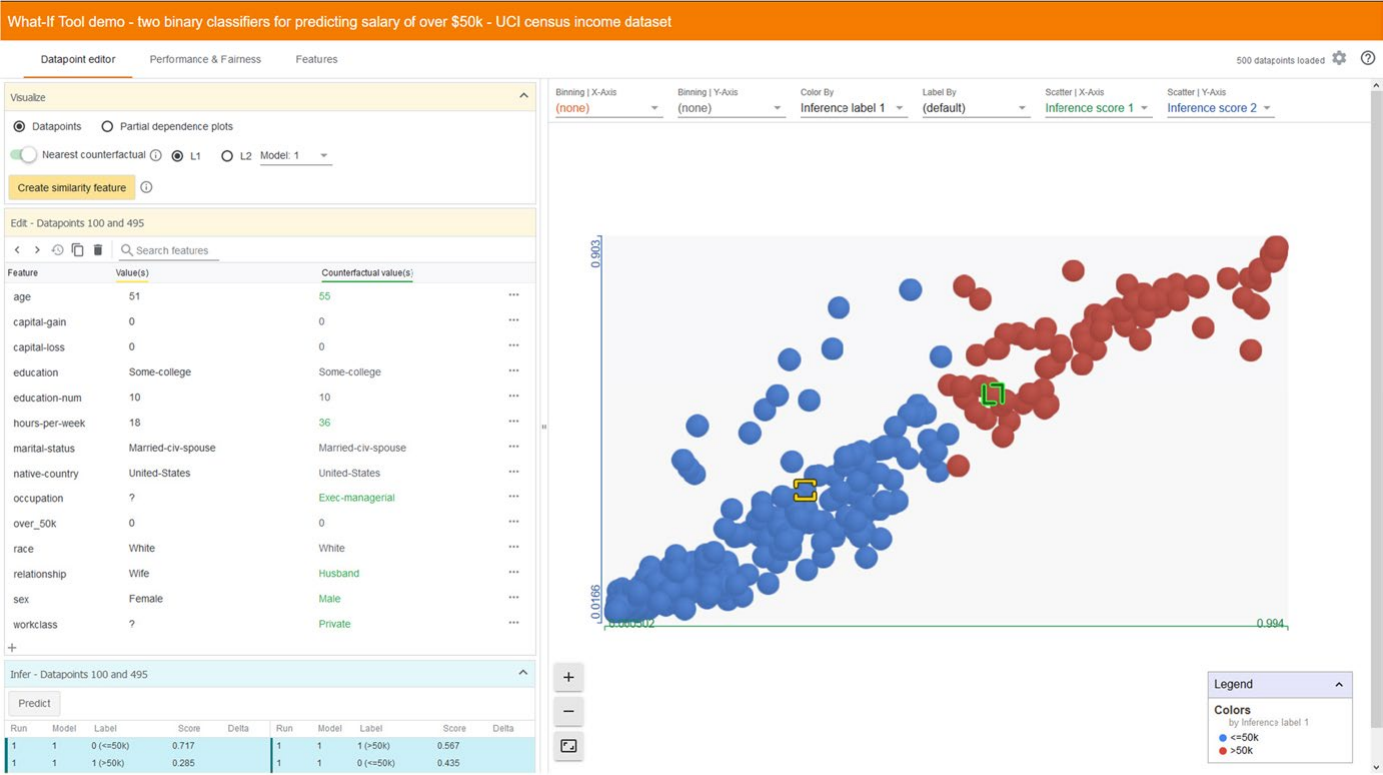
Finding the nearest counterfactual with the What-If tool (Google) (UCI census income dataset). Source: https://pair-code.github.io/what-if-tool/demos/uci.html

While ML took off in the 1990s with algorithms producing models that often could easily be explained (such as regression and single decision trees), soon enough the modelling became more complex (such as boosting and bagging, neural networks, deep learning); accordingly, interpreting models was no longer possible. How to interpret black boxes and the predictions they generate, both in general and for individual datapoints? Concerning this issue of explainability,^39^ the three companies have put in great efforts as well (listed in Table 3). In their documentation materials, Google, Microsoft, and IBM all stress the point—accepted wisdom by now—that explanations must be tailored to the specific public involved: whether data scientists, business decision-makers, bank clients, judges, physicians, hospital patients, or regulators. Each of these groups has their own specific preferences for what an explanation should entail.^40^ With this in mind, Google has further developed their What-If tool (already mentioned above).^41^ It enables ML practitioners to focus on any particular datapoint and change some of its features manually in order to see how the outcome predicted by their specific model changes. In particular, one may find the most similar datapoint with a different prediction (‘nearest counterfactual’) (cf. Fig. 3). Moreover, one can explore how changes in a feature of a datapoint may affect the prediction of the model (partial dependence plots).

In comparison, the options developed by Microsoft and IBM are more extended. Their packages each have their own flavour. Let me first discuss some IBM tools, bundled in their AI Explainability 360 Toolkit.^42^ As directly interpretable models, they offer the BRCG (Boolean Rule Column Generation) which learns simple (or/and) classification rules, and the GLRM (Generalized Linear Rule Model), which learns weighted combinations of rules (optionally in combination with linear terms). A more experimental tool for obtaining an interpretable model is TED (‘teaching AI to explain its decisions’), which allows you to build explanations into the learning process from the start. Post-hoc interpretation tools are also made available. For one, several varieties of ‘contrastive explanations’: identification of feature values that minimally need to be present for a positive outcome, in combination with the relative importance of those features (‘pertinent negatives’). Similarly, their ‘Protodash’ method allows to put a specific datapoint under scrutiny and obtain a few other datapoints with similar profiles in the training set (prototypes), thus suggesting reasons for the prediction produced by the model. As can be seen, IBM offers tools that are similar to the What-If tool from Google, but they unfold a much broader spectrum of approaches.

**Fig. 4:**
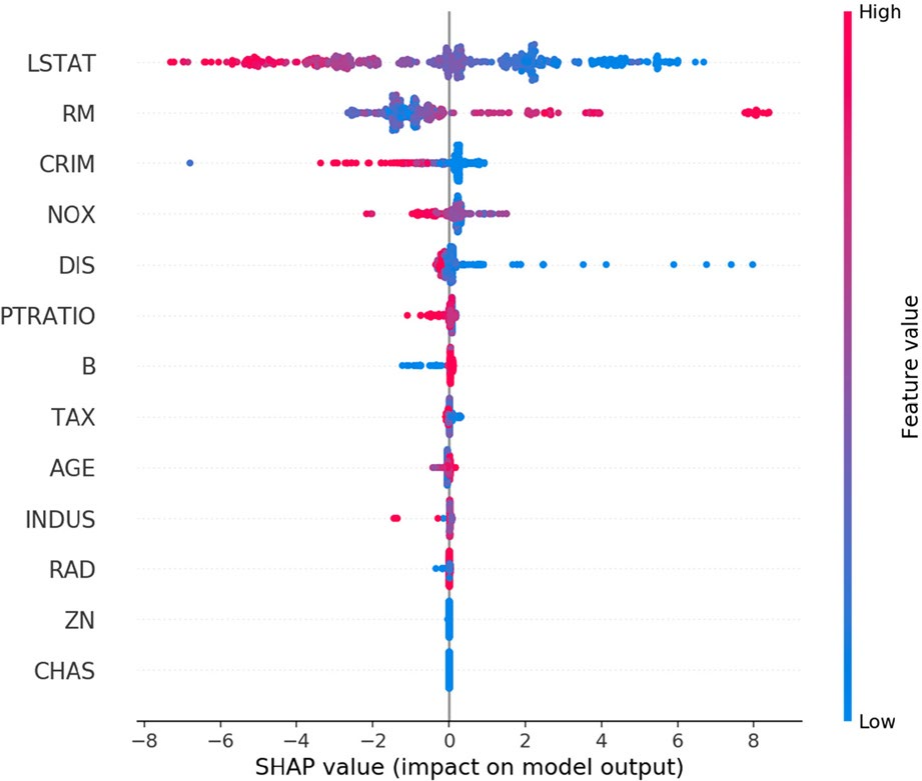
Feature values and their impact on model output (SHAP value); high feature values in red, low feature values in blue (global explanation). Source: https://github.com/slundberg/shap

**Fig. 5:**
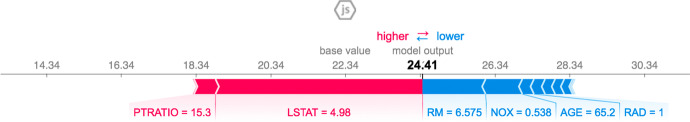
SHAP force plot, showing the contribution of features pushing model output higher (red) or lower (blue) than the base value (local explanation). Source: https://github.com/slundberg/shap

Microsoft, finally, has also developed a variety of interpretability tools (under the label InterpretML), which, again, are based on advances in ML as recently published in computer science journals.^43^ As an interpretable model, they propose the novel Explainable Boosting Machine (yielding both accuracy and intelligibility). For black box ML models, several explainer tools are provided. First and foremost, a family of tools is offered based on SHAP values—the method, pioneered by Lloyd Shapley, originates in game theory. The approach is model-specific, so for each type of ML model, a separate explainer has to be encoded. A SHAP-based explainer may then contribute to a *global* explanation by showing the top important features and dependence plots (the relative contribution of feature values to feature importance) (see Fig. 4). The explainer can also produce *local* explanations by calculating the importance of features to individual predictions (see Fig. 5) and by offering so-called what-if techniques that allow to see how various changes in a particular datapoint change the outcome.

Secondly, besides SHAP explainers, a new interpretable model may be trained on the predictions of a black box model. Either train an interpretable model (say linear regression or a decision tree) on the output data of the black box model under scrutiny (global surrogate model; the method is called ‘mimic explainer’) or use the LIME (Local Interpretable Model-Agnostic Explanation) algorithm to train a local surrogate model that ‘explains’ an individual prediction.^44^ Finally, for classification and regression models, the ‘permutation feature explainer’ is provided. This global explanation method revolves around the idea of randomly shuffling datapoint features over the entire dataset involved.

#### Google, Microsoft, IBM: Security and Privacy Tools

Before elaborating on the tools developed by Google, Microsoft, and IBM for enhancing security and privacy in AI, let me first briefly explain what these concepts imply for AI specifically. Whenever sensitive data are collected and processed by organizations, privacy is a vital issue of concern. The usual tools to handle such concerns are encryption, anonymization, and the like. Organizations have been confronted with this issue for quite some time now; the same goes for security. If sensitive data are involved in ML applications in particular, *additional* issues impacting on privacy and security come to the fore since adversaries may mount ‘adversarial attacks’ on the system. Such attacks aim to get hold of system elements (the underlying data, the algorithm, or the model) or to disrupt the functioning of the system as a whole. These issues are of more recent date, and efforts to deal with them are still in their infancy.

Several adversarial attacks may usefully be distinguished.^45^ In the first category (targeting system elements), attackers may retrieve at least some additional feature values of personal records used for training (‘model inversion’). Similarly, outsiders may infer from a person’s record whether he/she was part of the training set (‘membership inference attack’). After a client has used data to train an algorithm, these may be recovered by a malicious provider of ML services if he/she has installed a backdoor in that algorithm (‘recovering training data’). Attackers may even emulate a trained model as a whole, by repeatedly querying the target (‘model stealing’).

The second category concerns the integrity of an AI system as a whole. Attackers may produce an ingenuous query and submit it to a model in deployment in order to disturb its classification performance (‘perturbation attack’, ‘evasion attack’). Specific data to be used for training may be compromised (or even datasets as a whole that are widely in use poisoned), affecting the trained model’s performance (‘data poisoning’). Finally, training may be outsourced to a provider that tampers with the training data and installs a backdoor in the produced model, resulting in degraded classification performance for specific triggers (‘backdoor ML’).

All this is very new terrain, with lots of active R&D, but as yet only a small repertoire of solutions and remedies. Most prominent is the approach of generating adversarial examples and retraining one’s algorithm to become immune to them (‘adversarial ML’). Another approach is ‘differential privacy’, which adds noise to ML: either locally to datasets so that individual datapoints can no longer be identified by users of the datasets or, in a more sophisticated vein, to the actual *process* of ML itself in order to render the final model privacy-proof—the algorithm becomes ‘differentially private’. The model no longer ‘leaks’ training data as belonging to specific individuals.

Finally, ‘federated learning’ is on the march.^46^ Suppose an ML model is to be trained dynamically from local data on mobile phones. These are no longer uploaded to a central location but stay where they are. After receiving the current model, each phone locally performs an update which is uploaded and used for ‘transfer learning’. Apart from enhancing security, this obviously reduces the risk of violation of privacy.

Which tools of the kind are offered by Google, Microsoft, and IBM (see Table 3)? IBM appears to be the minor player here. It only offers the Adversarial Robustness 360 Toolbox, an open sourced toolbox with tools to defend deep neural networks against adversarial attacks.^47^ Microsoft, on their part, have open sourced the WhiteNoise toolkit for implementing differential privacy schemes, focussing on datasets.^48^

**Fig. 6:**
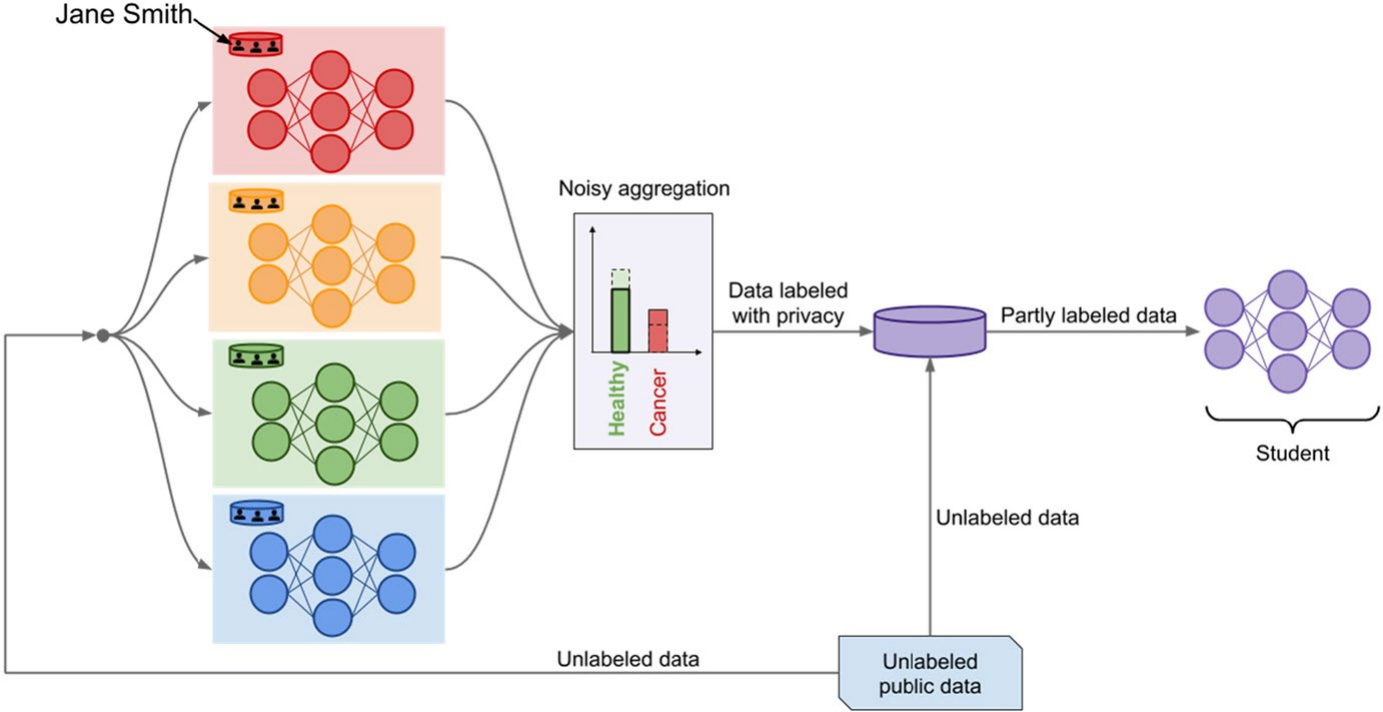
Teacher-Student Adversarial Training (Google). Source: http://www.cleverhans.io/privacy/2018/04/29/privacy-and-machine-learning.html

Google, finally, seems to have the edge at the moment. To begin with, they offer a library of adversarial attacks (CleverHans).^49^ Furthermore, they have developed two differential privacy schemes focussing on the very process of ML. The first scheme, TensorFlowPrivacy, provides a method that introduces noise into the gradient descent method used in neural networking.^50^ Private Aggregation of Teacher Ensembles (PATE), the second scheme, proposes ML in two steps.^51^ Teach an ensemble of models first, add noise to their collective ‘voting’, and learn a student model from fresh data (which have obtained their label from the ensemble voting). Only the latter student model is to become public, all others are discarded (see Fig. 6). Their Cobalt pipeline, finally, combines various security measures: federated learning, local differential privacy, and anonymization and shuffling of data.^52^

#### Accountability Tools

The final aspect that has frequently been stressed as an element of responsible AI is accountability (cf. Table 2): providers of AI have to be able to produce a proper account of all the steps in the process of production of their solutions. While most companies on my list of 24 just pay lip service to this requirement, only three firms have actually developed accountability tools tailored to AI. In a kind of silent competition, Google, Microsoft, and IBM have each submitted proposals of the kind (Table 3). As we will see, these complement each other. Let me first discuss the proposal from (mainly) Microsoft employees about datasets to be used for training (Gebru et al. 2018–2020). The authors emphasize that quality of datasets is paramount for ML. Datasets may contain unwanted biases, and the deployment context may deviate substantially from the training context. As a consequence, the trained model performs badly. In order for dataset consumers to be prepared, they suggest that dataset creators draft ‘datasheets for datasets’. These are to give full details about how the dataset has been ‘produced’: its creation (e.g., by whom), composition (e.g., labels, missing data, sensitive data, data splits), collection (e.g., sampling procedure, consent), pre-processing (e.g., cleaning, labelling), uses (e.g., in other instances), distribution (e.g., to whom, licensing conditions), and maintenance (e.g., support, updates).

Further, ‘model cards’ are proposed by Google employees (Mitchell et al. 2018–2019). These cards provide details about the performance of a specific trained model. Subsections are to specify details of the model; intended uses; factors to be considered for model performance; appropriate metrics for the actual performance of the model; specifics about test data and training data; and ethical considerations to be taken into account. As the authors note, such cards are especially important whenever models are developed, say by Google, and offered in the cloud (‘Google Cloud’) and subsequently deployed in contexts such as healthcare, employment, education, and law enforcement (Mitchell et al. 2018–2019: 220). From the point of view of responsible AI, this accounting procedure is interesting, since any of its aspects can be incorporated in a model card as an issue to be reported on. Take fairness (Mitchell et al. 2018–2019: 224): whenever different groups (say age, gender, or race) are involved, the actual performance of the model across them can be specified (say by means of a confusion matrix). This allows to inspect whether equal opportunity has been satisfied.

Finally, ‘Factsheets for AI-services’ are proposed—by IBM employees this time (Arnold et al. 2018–2019). As a rule, AI services do not rely on single datasets or single pretrained models alone but on an amalgam of many models trained on many datasets. Typically, consumers send in their data and just receive answers in black box fashion. In order to inspire confidence in such services, the proposed factsheets can first of all enumerate details of the outputs, training data and test data involved, training models employed, and test procedures adhered to. Furthermore, several issues related to responsible AI may be accounted for: fairness, explainability, and security/safety (such as protection against adversarial attacks and concept drift).

### External Collaboration and Funding concerning Responsible AI Research

Many companies on my list of 24 perform some degree of R&D internally. Moreover, some of them nurture close ties with outside non-profits or universities concerning research areas they deem important. Regular publications in computer journals are common. Take DeepMind. Acquired by Google in 2014, it is now Google’s research department in the UK that cooperates with many outside organizations and has maintained a consistent research output over the years. Or take IBM. Big Blue has always had a strong research department, outside collaborations, and a high research output as a result. At the moment, it does joint R&D with dozens of academic institutions. One recent example, initiated in 2019 (see Table 3), is their joint research with the Institute for Human-Centred AI at Stanford, focussing on responsible AI—as well as natural language processing and neuro-symbolic computation.^53^

As far as responsible AI is concerned, a new pattern has emerged on top of these regular R&D efforts: erecting completely *new institutes* (or programmes) with an exclusive focus on responsible AI and furnishing the money for them. Three such initiatives are current (Table 3). Facebook has created the Institute of Ethics in AI, located at the TU Munich (2019). With a budget of 7.5 million dollars over five years, it will perform research about several aspects of responsible AI.^54^ Amazon co-finances an NSF project called ‘Fairness in AI’ with 10 million dollars over the next three years (2019). The project distributes grants to promising scholars located at universities all over the USA. Its title is slightly misleading, though, since they actually intend to cover many aspects of responsible AI, not just fairness.^55^ IBM tops them all by funding a separate Tech Ethics Lab at the University of Notre Dame (2020). With 20 million dollars at their disposal for the next 10 years, the lab will study ethical concerns raised by advanced technologies, including AI, ML, and quantum computing.^56^

For a proper perspective, these initiatives should be placed in context. For IBM, it clearly represents an extension of already considerable research into ethical aspects of AI/ML. For Facebook and Amazon, the context differs: their programmes mark an effort to *catch up*. Their spending on research into aspects of responsible AI (and corresponding publication output) has been negligible until recently—especially in comparison with the main companies pushing for responsible AI: Google, Microsoft, and IBM.^57^

## Implementation of AI-principles: Overview and Discussion

Let me at this point summarize the steps which the 24 companies that committed to principles for responsible AI have actually taken to move from those principles to their implementation in practice. The summary will be used for an attempt at answering the question whether we can accuse the companies involved of mere ‘ethics washing’ or not.

### Overview

As appropriate governance structure for responsible AI, it is usually suggested to introduce a two-fold setup at the top of an organization: an ethics advisory group (or council) for input from society and an ethical committee (or review board) for guiding and steering internally towards responsible AI. It turns out that a large majority on my list of 24 did not care about such new governance: only eight companies did introduce such measures. Five firms installed an ethical committee alone, while three firms installed the full governance setup of ethical committee *and* advisory group. Remarkably, none of the largest companies usually very vocal about their mission to realize responsible AI (Google, Microsoft, and IBM) cared to install such an advisory council on top of their ethical committee.

The statistics are slightly better as far as developing new educational materials for training purposes (blog entries, guidelines, checklists, courses, and the like), or coding new software tools is concerned. While half of all 24 committed firms did not contribute anything of the kind, four companies introduced relevant training options, and three companies incorporated some smaller tools (for fairness or explainability) into their current software offerings. Only five companies realized that fully responsible AI can only come to fruition if documentation and software tools about all aspects of responsible AI are created and made available to employees/clients.

Amongst these, Google, Microsoft, and IBM stand out. Their educational materials and software tools are in a class of their own that no other large company—such as Amazon or Facebook—has been able to match. Based on thorough research, usually performed together with other researchers at universities and non-profits, the whole package represents the forefront of current ideas about responsible implementation of AI. Let me just recapitulate some highlights.

Concerning fairness, a whole menu of metrics has been developed. Building on this, several techniques for mitigating bias have been developed, each implemented in relevant source code. As far as explainability is concerned, post-hoc interpretable models can be learned on the predictions from a black box model. Methods have been developed to find the most similar datapoints with the same prediction or the nearest datapoints with the opposite prediction. Approaches based on SHAP values report the most important features for either a model as a whole or a particular prediction. For countering adversarial attacks, prominent tools are libraries of adversarial attacks and the addition of noise to datasets or to the neural networking itself. Finally, serious steps towards realizing accountability are schemes that enable to account for datasets, models, or AI-as-a-service.

### Discussion

So, first steps towards responsible AI have been taken, in particular, by the largest tech companies involved. How are we to evaluate these steps? Do they amount to mere ‘ethics washing’? The charge of mere ethics washing is to mean that all the developments reported above are just ‘ethical theatre’ (yielding nothing of value) intended to keep regulation at bay (a goal that may or may not be reached).^58^ Such activities may substitute for stricter regulation. In order to inspect this charge, I propose a nuanced approach which breaks it down into its component parts and discusses them in an analytic fashion. Building upon arguments developed by Elettra Bietti, Brent Mittelstadt, Julia Powles, and others, three perspectives are explored, focussing, respectively, on (a) the impact on regulatory alternatives, (b) the constraints on governance initiatives within a firm, and (c) charges of a narrow focus on ‘technological fixes’.

#### Impact on Regulation

Concerning regulation, the actual impact of new governance structures and new educational materials and software tools (combined with massive funds for collaboration between industry and academia) (Table 3) on regulatory alternatives is to be explored, as well as the companies’ intentions behind these actions. First, did these initiatives for responsible AI effectively freeze regulatory alternatives? Has valuable time been won by the companies involved and regulatory pressures staved off (cf. Bietti, 2020: 217)? I would argue that this potential effect has not materialized. In retrospect, after some early sporadic calls for responsible AI (from 2016 onwards), the flood of declarations of AI principles issued by companies began in earnest at the beginning of 2018. This soon enough led a dozen of them to initiate experiments with new governance structures and/or creating manuals and software tools for responsible AI (as presented in Table 3). In addition, important industry-academia collaborations were staged (Table 3). Concurrently though—with small beginnings even before 2018—governmental and civil society actors, the professions, and academia alike started to press home their views on responsible AI. In this cacophony of voicesm important corporations such as Microsoft, IBM, and Google (in that order) began to realize that governmental regulation of AI was unavoidable. From mid-2018 onwards, they publicly uttered their willingness to cooperate with efforts towards regulation of the kind (to be discussed more fully below, Sect. 5.3.2). So, any softening or delaying of AI regulation does not seem to have occurred.

As to their intentions, second, some companies committed to AI principles (from Table 3) initially may well have harboured hopes for state regulation to be delayed or weakened as a result of their AI initiatives. After all, such hopes are usually supposed to be the intention behind pleas for self-regulation by firms (cf. also Sect. 5.1 below)—and the campaign for responsible AI as just described is just another form of self-regulation, this time *within* the individual firm.^59^ Anyway, whatever hopes may have been entertained by any firm; these were effectively squashed by the incessant pressures from society for an AI responsive to its needs.

#### Corporate Constraints

Next, one has to consider the corporate context within which these initiatives for responsible AI unfold, potentially reducing the scope of possible reform (cf. Bietti, 2020: 216–217). Several questions impose themselves. What discussions are considered legitimate? What is considered out of bounds? How do decision powers influence outcomes? How does the new governance for responsible AI influence actual project decisions? Were the AI products developed demonstrably more ‘responsible’? Were any projects deflected in their course? Were any projects (say about autonomous AI) halted out of ethical considerations? Are AI governance practices made transparent? May outside experts speak freely about their experiences or not?

Answering these questions about the gains of corporate new governance of AI as tabulated in Table 3 is a thorny issue. Concerning ethics councils, review boards, and team diversity, I have only assembled materials as published by the firms themselves. This produces an overview of rules and procedures that have been introduced—not of the results obtained (if any). Companies do not report detailed evaluations of the various procedures involved. So, I simply cannot answer the above questions.

Occasionally, incidents leak out that remind us that company preferences and constraints are in force. In spite of their lofty AI principles, Google had initiated cooperation with the Pentagon for the Maven project; their task was to improve the analysis of footage of surveillance drones by means of ML. When details of this contract came into the open, massive employee protest erupted. As a result, the company decided to cancel its cooperation (mid-2018).^60^ More recently (December 2020), AI researcher Timnit Gebru was fired by Google.^61^ The direct cause was an argument about the future publication of a research paper she had co-authored. It alerted to the dangers of large natural language modelling (like BERT and GPT-3), especially the large environmental footprint it requires.^62^ In the background, though, there was also resentment on the part of Google management about her fight for inclusiveness inside the company. With this alarming incident also, several thousands of people (including many Googlers) immediately protested. Doubts about Google’s stance towards inclusivity and principled AI were expressed openly. Another prominent member of the AI ethics group, Margaret Mitchell, who openly supported Gebru, was fired two months later (February 2021).^63^ Steps the company might take in response to the protests and actual repercussions for the company’s efforts towards responsible AI as a whole are yet to be determined. Have responsible AI and Google’s corporate environment become incompatible after all?

Obtaining more thorough insights about the achievements of said new governance would require in-depth scholarship that obtains independent access to the firms, their employees, and their committee members. So, the only conclusion that can be drawn for the moment is that, indeed, corporate energies have been channelled into bending AI practices towards more ‘responsibility’—with research participants apparently trying to achieve tangible outcomes. Whether this is actually the case remains to be determined.

#### A Narrow Focus on ‘Technological Fixes’?

The final fruits of responsible AI efforts are the courses, materials, guidelines, instructions, and software packages as tabulated in Table 3. Several authors have been dismissive of these steps on the road to responsible AI practices, the new software tools in particular. These are variously debunked as ‘technological solutionism’, as based on the mistaken conception of ethical challenges as ‘design flaws’ (Mittelstadt, 2019: 10); as ‘mathematization of ethics’ (Benthall, 2018) which only serves to provide ‘a false sense of assurance’ (Whittaker et al. 2018: ch. 2, p. 27); or, as Julia Powles puts it, talking about the efforts to overcome bias in AI systems: ‘the preoccupation with narrow computational puzzles distracts us from the far more important issue of the colossal asymmetry between societal cost and private gain in the rollout of automated systems; (…) the endgame is always to “fix” A.I. systems, never to use a different system or no system at all’ (Powles & Nissenbaum 2018).^64^

The charge is that the AI community of experts is tempted to reduce the ethical challenges involved to a technocratic task: the appropriate conceptions are to be made computable and implementable, and all AI will be beneficial. As a result, the inherent tensions behind these ‘essentially contested concepts’, emanating from the clash between the interests of the various stakeholders involved, are ignored and remain unaddressed.

Note the parallel: while the earlier critique of new instruments in general for responsible AI argued that these may obscure regulatory initiatives and thereby soften or keep them at bay, this *additional* critique of technical solutions in particular argues that these tend to obscure the more fundamental problems underlying application of AI and therefore keep consideration of them at bay. Both may be considered to be forms of the ‘ethics washing’ argument—but they point to different phenomena being obscured, both deemed a nuisance by companies.

How serious is this technocratic critique to be taken? It is no coincidence, of course, that in their declarations, the 24 committed companies have precisely zoomed in on those principles that require new *technical* methods (for fairness, explainability, security and privacy). To them, as computer scientists, it *is* appealing to solve the puzzles involved. However, the charge appears to suggest that, therefore, close to nothing has been gained. I dare to challenge this assessment.

Take explainability: of course, only a broad societal debate can determine what a proper explanation is to mean, for each relevant public and for each relevant context. But let us not overlook the fact that that debate is already underway. Academia and industry have been discussing the need to distinguish between different publics that require explanations, and the sort of explanations they require. Thereupon, much energy has been put into translating these requirements into concrete software tools (most of which have been open sourced). As a result, the ethical discussion has not necessarily been foreclosed, but has acquired the tools needed for the debate to continue and possibly reach some kind of consensus. Instead of decrying the ‘mathematization of ethics’, one could try to see it in reverse: the mathematical tools created may readily invigorate the ethical debate.

The same argument can be made for fair AI. The concept of fairness is essentially contested, indeed. But for now, a debate spanning academia and industry circles (and beyond) has made clear that a multitude of fairness conceptions are to be distinguished. As the next step, this has been translated into more precise fairness metrics, which subsequently have been implemented in software tools (open sourced again) that allow updating learned models to conform to one’s fairness metric of preference. Again, I interpret this development as a welcome tool for a fruitful ethical discussion, not as a technocratic solution that necessarily diverts attention away from the underlying societal tensions and stakeholders involved.

Rounding off the whole discussion about ‘ethics washing’ in this Sect. (4.2), I conclude that overall, some progress towards responsible AI has been made. Although an assessment of the new governance for responsible AI remains elusive, some positive first steps have been taken, especially by the companies on my list that have produced guidelines, brochures, checklists, and, last but not least, concrete software implementations for the newly invented techniques (Table 3).^65^ For those companies at least, the accusation of being involved in mere ‘ethics washing’ seems to be misplaced. As for the committed companies on my list that apparently—at least publicly—have not set any first steps on that road, the jury is still out; though belatedly, they still might catch up.

### A Future with Responsible AI?

So, may we conclude that a future with responsible AI is near and all the promises will turn into reality? That is, presumably the following scenario unfolds. The responsible AI tools from Google, Microsoft, and IBM will (continue to) trickle down and increasingly be used by other producers and consumers of AI solutions. As a result, biases will be reduced to a minimum, and recipients at the end of the AI chain (such as physicians, patients, or bank clients) and overseers (such as regulators) will receive the explanations they desire—all of this (almost) completely shielded from the fall-out of adversarial attacks. I am afraid, however, that it is too soon for jubilation; many obstacles remain to be overcome.

Let us consider, by way of example, the companies in my research that did introduce training materials and/or software tools connected to *explainability*. For one thing, it is difficult to ascertain whether their training sessions for ML practitioners to develop the correct ‘mindset’ for handling the issue of explainable AI are actually effective. Moreover, whenever companies do use the software tools involved, overwhelmingly they appear to be used by ML practitioners only for the purpose of sanity checks on the models they produced (Bhatt et al. 2019–2020).^66^ The relevant end users simply do not (yet) receive explanations from them.

Studies suggest that many hurdles have to be overcome before ML practitioners feel confident to do so (Bhatt et al., 2020). There are some technical issues (such as how to find counterfactuals and construct measures of confidence). More importantly, though, explanations have to be put into the particular context, stakeholders’ needs have to be considered, and the process of explanation should preferably allow interacting with the model. Finally, most difficult of all, interpreting important factors of an explanation as causal remains a fragile undertaking. The article by Bhatt and others (2020) provides a fascinating array of situations in which unanswered urgent questions emerge—bringing home the point that technical prowess concerning ML is one thing but putting those instruments to work in actual practice in responsible fashion is quite another. That implementation phase is a formidable hurdle that is underexplored at present.

Currently, only a fraction of companies (let alone organizations in general) are recognizing the risks associated with the explainability of AI and taking steps to mitigate them. A McKinsey study from 2019 amongst firms using AI found that the percentage was 19% (amongst ‘AI high performers’ it rose to 42%).^67^ If a much wider audience of companies (and other organizations) becomes convinced that the call for explainable AI must be answered, they will have to adopt the relevant training materials and software tools. That spread, though, is likely to face additional obstacles. Kaur et al. (2020) did research about the potential use of the interpretable GAM (General Additive Model) and SHAP explainers by data scientists. After having been introduced to the new tools, the majority of their respondents did not appear to properly understand the tools and their visualizations. As a result, instead of (correctly) using them for critical assessment of their models, these practitioners either uncritically accepted the tools (‘overuse’), blinded by their public availability and apparent transparency, or they came to distrust those tools and showed reluctance to use them at all (‘underuse’). If these obstacles are not cleared, expectations about proper explanations being provided to end-users are even more utopian.

So, the road to AI with stakeholders being satisfied in their demand for explanations seems to be full of obstacles. The same goes, I presume, for the road to fully de-biased, fair, and secure AI—each with obstacles of their own. And as concerns the accountability tools mentioned, these may evolve into standards, but as long as they remain voluntary, their wide and—especially—faithful and complete adoption is far from guaranteed.

After these sobering conclusions about the future of (responsible) AI, it is time to move on to the issue of AI regulation.

## Regulation of AI

Until now, I have been focussing on the 24 committed companies individually and their efforts to implement responsible AI. However, the issue of such AI is also hotly debated in society at large, as the numerous declarations about AI by a range of societal organizations attest to. Are the committed firms willing to grant society and its constituents a say over affairs of (responsible) AI? Phrased otherwise, what are their attitudes to regulation of AI by societal actors at large?

**Fig. 7:**
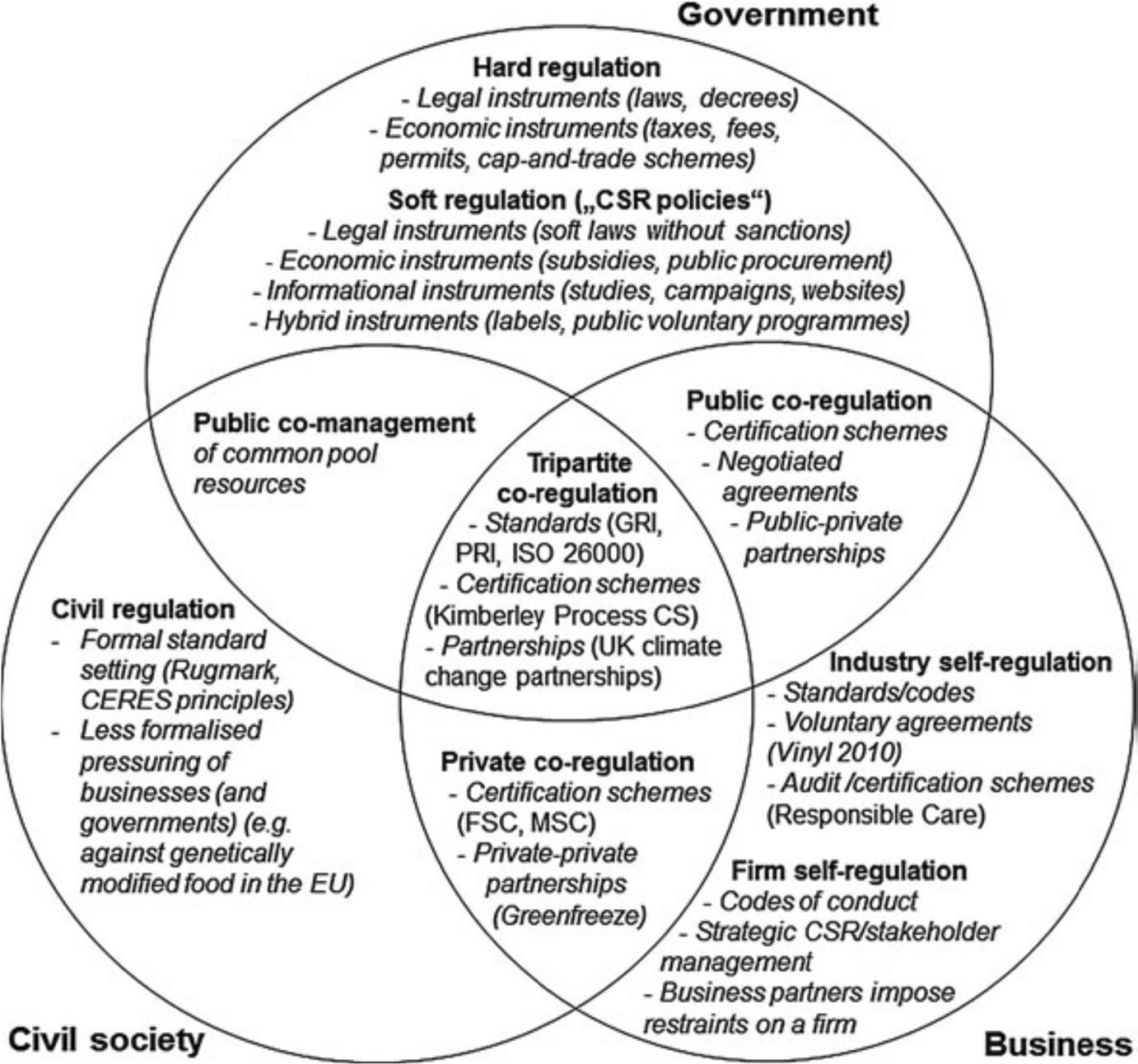
Domain-specific types of regulation, as well as domain-spanning types of co-regulation. Source: Steurer (2013): 398

Some explanation about the term regulation is in order at this point. Steurer (2013) constructed a synoptic view of the various possible forms of governance of business by state and non-state actors. He focusses on the various ‘actor constellations’ involving government, business, and/or civil society that constitute a variety of modes of regulation.^68^ While confining myself to those parts of his analysis that are useful for my purposes, let me first introduce the three basic types of regulation (see Fig. 7). The first is *self-regulation* by businesses (Steurer, 2013: 394–396). This can take place at the level of the individual firm—which has been discussed extensively above. It can also take shape at the industry level, in sectors which are relevant. Firms (or their trade associations) cooperate in the pursuit of developing best practices, codes of conduct, standards, or audit schemes, which companies subsequently may adhere to on a voluntary basis. A large pool of standardization bodies across the globe offers their services for the purpose. Noticeably, the ‘shadow of hierarchy’—that is, governmental intervention—is usually not far away when such ‘voluntary’ initiatives are unfolding (cf. Steurer, 2013: 399–400).^69^

The second basic type presented by Steurer is *regulation by government—*usually referred to as regulation tout court (Steurer, 2013: 393–394). Although governments usually issue laws, decrees, or directives that steer the issues at hand (‘hard regulation’), they may also use economic instruments (such as taxes or fees), or publish guidelines, brochures, or reports that suggest the correct course of action (‘soft regulation’). The third and last basic type is *regulation by civil society* and its organizations (Steurer, 2013: 396–397). These include organizations such as Amnesty International and Greenpeace; in a wider sense, they are often joined by critical investors, critical employees, and critical consumers. These may develop pressure upon companies or confront them in direct ways (blockades, boycotts) in order to have them accept specific standards (e.g., concerning child labour) or adopt a specific course of action (such as abandoning the production of genetically modified food).

Besides these ‘one-sided’ forms of regulation, in which the sphere of business is steered by a power sphere outside it, Steurer distinguishes modes in which spheres are *jointly* establishing a regulatory regime. This can be realized in two distinct ways. On the one hand, actors may join forces and resources in non-confrontational ways (*co-regulation*): government and business, or civil society and business cooperate as ‘partners’—to be referred to as public or private co-regulation respectively (Steurer, 2013: 396–397; cf. Fig. 7). Examples include jointly developing new standards (e.g., for sustainable coffee) or certification schemes (e.g., for sustainable forest management).

On the other hand, societal domains—government and business in particular—can end up working together on regulation in confrontational ways. According to Steurer (2013: 401), this yields a novel form of regulation imbued with an air of antagonism that fuses governmental regulation and self-regulation into a new *hybrid*. Such a hybrid regulatory regime is very common in Europe, and a variety of forms can be found in practice. These may usefully be classified along two variables: whether they are explicitly mandated by the state or implicitly suggested, and the policy stage in which public involvement takes place: the early stage of rule-making, or the later stages of implementation, monitoring, and enforcement (combined) (cf. Senden et al., 2015; in particular, graph 2, p. 36). A prominent example is ‘enforced self-regulation’: the state commands firms to develop a self-regulatory regime while retaining the right to monitor and sanction the results. Such subcontracting clearly exhibits the characteristics of both state regulation and self-regulation.^70^ Another well-known hybrid example is ‘responsive regulation’, a term coined by Ian Ayres and John Braithwaite two decades ago (Steurer, 2013: 401): laws are combined with a repertoire of tools for the regulator, ranging from persuasion up to sanctions, in order to elicit compliance from and stimulate self-regulatory activities of firms under regulatory scrutiny.

With this conceptual framework at hand, I performed a web search in order to find out what the 24 committed companies have published on this topic of regulation of AI. What are their thoughts and actions about self-regulation (at the industry level), civil regulation, and state regulation of AI (cf. Appendix on search method, searching with KS3)?^71^ Notice that for the committed companies in this research AI has effectively morphed into ‘responsible AI’ — to them, there is no AI if not responsible. As a result, their proposals for proper regulation of AI refer to AI that is ‘responsible’ by default. The heated discussions about facial recognition software, for example (cf. Sections 5.2, 5.3.2, and 6 below), exemplify that at present, regulatory proposals for AI are invariably imbued with principles for responsible AI.

### Self-Regulation of AI at the Industry Level

Let me first remark that AI companies participating in the Partnership on AI (17 in all) may be considered to subscribe to the development of appropriate *best practices* for AI. After all, developing such benchmarks for AI is the raison d’être of the PAI. On their website, an impressive list of reports and research papers of the kind may be consulted.^72^ Remarkably, the Partnership started off as a self-regulatory exercise at the industry level, but soon enough *other* stakeholders were invited and joined as partners. As a result, the initiative turned into a ‘private co-regulation’ arrangement (cf. Section 5 above), a cooperation between industrial and civil society members as equal partners in search of best practices.^73^

From my search of documents and statements produced by all 24 committed AI companies, several other more pronounced self-regulatory instruments for responsible AI came to the surface (Table 4). Most often (by 12 of them), the development of adequate *standards* for AI was recommended. When they appear in their final form, companies may, on a voluntary basis, request to be certified for their adoption. These standards are sometimes specified as global standards (by Accenture, Element AI, Facebook, Google, and Intel), sometimes as domestic standards (Canadian, by Element AI; European, by Tieto). Moreover, the organizational setting that is to develop the standards may specifically be mentioned: NIST (by Amazon), IEEE (by Accenture), or an industry-driven standard-setting organization (by McKinsey, Telefónica). Firms in the medical sector (Health Catalyst, Philips) obviously stress the need for medical standards—without them, no AI product can be launched in that sector at all.

**Table 4:** Companies practicing and developing AI in order to apply/sell/advise about AI that explicitly have committed to principles or guidelines for AI and/or to the tenets for AI of the Partnership on AI—tabulated according to their attitudes towards self-regulation of AI and towards governmental regulation of AI

AI companies committed to AI principles^1^	Attitude towards self-regulation of AI^2^	Attitude towards governmental regulation of AI^3^
Amazon	Standards (together with NIST)	Need for regulation of facial recognition software
Google	Global standards	Need for regulation of AI, such as self-driving cars and drones; support for GDPR and moratorium on facial recognition software; approach to be proportionate to risks
Microsoft	Standards	Need for new regulation of specific AI applications, such as privacy and facial recognition; support for US bills of the kind
IBM		Need for ‘precision regulation’ of AI: focus on specific applications like facial recognition or illegal online content; rules to be proportionate to risks; standardization bodies to be designated by governments
Intel	Global standards (privacy)	Need for regulation of privacy
Facebook	Global standards	Need for regulation of privacy (à la GDPR) and of harmful online content (cf. Oversight Board)
Telefónica	Best practices and standards to be developed by industry partnerships	Need for smarter regulation of AI: not ex ante but ex post (regulatory ‘sandboxes’); digital platforms should be regulated
Accenture	Best practices, global standards, several codes of ethics (concerning data and data scientists)	
SAP	Global code of conduct for good AI business practices	No need for new legislation for AI
Philips	Medical standards	
Salesforce		
McKinsey (Quantum Black)	Best practices; automotive industry to proactively shape standards	Advocates engagement of firms with regulation of AI in automotive sector, healthcare, and education; advocates regulatory sandboxes
Sage		
Tieto	European standards	Need for regulation of AI: weigh risks against benefits, consider concrete use cases, allow regulatory sandboxes; regulation of biometric identification, must not hinder innovation; regulation of the use of personal data, opt-out instead of opt-in
Health Catalyst	Medical standards	
Deep Mind (Google subsidiary)		
Element AI	Domestic and global standards	

^1^Companies are ordered by revenue. Apple, Samsung, Deutsche Telekom, Sony, Kakao, Unity Technologies, and Affectiva have been omitted from the table since my searches yielded no results for them.

^2^Note that members of the PAI, even without explicit declarations of the kind, may implicitly be considered to be in favour of developing ‘best practices’ for responsible AI since that is the core of PAI activities.

^3^Proposals about governmental regulation of AI refer to documents published by the firm in question or to participation in such activities—occasional statements made in interviews, public appearances, and the like—as a rule are not taken into account.

Note: The sources that I drew my information in the table from are for the most part given in footnotes in the text of the article; otherwise, the sources are available on request.

Another tool of self-regulation, *ethical codes*, came to the fore sporadically. SAP advocates the development of a global code of conduct on ethical AI business practices which firms can sign up to.^74^ While a global code is preferred, there might also be arguments for creating a European code or an industry-specific code. The consultancy firm Accenture, on their part, though approvingly making mention of the long-existing ethical code for software engineering (jointly produced by IEEE and ACM),^75^ proposes the development of a new code for data ethics, as well as best practices for data sharing, at the level of the firm.^76^ More broadly, they suggest that organizations produce a code of ethics for the data science profession, to be adhered to by data scientists in general.^77^ This professional code may additionally inform codes tailored to a specific industry (healthcare, finance, etc.) or a specific organization.^78^

Let me observe after this tabulation of AI self-regulation efforts at the industry level that one might be tempted to extend the scope of the ethics washing argument (cf. Section 4.2, in particular note 58) to include such initiatives: are corporations only interested in industrial self-regulation as a mere façade that may possibly weaken or delay prospective governmental regulation? In fact, companies talking about best practices, standards, or codes of ethics usually have stricter state regulation at the back of their minds, as a threat to be avoided. The ‘shadow of hierarchy’ is never far away. The SAP initiative just mentioned for a global code of conduct is a case in point. As their report states: ‘A code of conduct could help address public concerns around AI and, as such, serve as a market-driven alternative to AI-specific regulation, which might hinder the development of the new technology’.^79^ The account of companies’ attitudes towards state regulation of AI below (Sect. 5.3.2 about the US firms in particular) will also bear this out: a preference for self-regulation of AI has been their default for long—until recent developments forced them to accept that proper regulation of AI could no longer be avoided. Nevertheless, although most often imbued with intentions to soften regulation, self-regulatory initiatives are not by definition to be interpreted as *mere* ‘ethics washing’—or more aptly as mere ‘standards washing’—since they may *also* create regulatory instruments, in this case best practices, standards, and ethical codes for responsible AI. These instruments have the potential to be effective in regulatory practice and cannot therefore be outright dismissed as just a façade.

After these findings about self-regulation, I will document the companies’ attitudes toward the other forms of regulation mentioned above—by civil society and by the state. Let me start with the former, less intrusive form of regulation.

### Regulation of AI by Civil Society

Before going into the attitudes of the 24 committed firms towards civil regulation of AI, let me first chart the civil forces that are actually pushing towards responsible AI. Non-governmental organizations outside industry have produced and disseminated a raft of declarations about the importance of responsible AI and their commitment to it. These statements represent the main voices of civil society.^80^ Prominent examples include the Toronto Declaration, the Universal Guidelines for AI, the Asilomar AI Principles, the Montreal Declaration, and the 10 principles for ethical AI from the UNI Global Union. Let me discuss each of them briefly.

‘The Toronto Declaration’ calls on both public and private sector actors to protect human rights in the age of AI, with a focus on equality, non-discrimination, and diversity.^81^ In the declaration, several other aspects of responsible AI such as transparency, explainability, and accountability are touched upon as well. It had been drafted in 2018 by Amnesty International and Access Now (a New York University based institute doing research about the social implications of AI). Further, the ‘Universal Guidelines for AI’ covers almost verbatim the aspects of responsible AI (as listed in Table 2)—plus some more obligations (e.g., for data quality and public safety) and prohibitions (on secret profiling and unitary scoring).^82^ These were formulated in 2018 by The Public Voice, a coalition set up in the 1990s to promote public participation in decision-making about the future of the Internet. Their goal is to bring civil society leaders and government officials together for ‘constructive engagement’. The ‘Asilomar AI Principles’ (2017), another prominent statement, covers most aspects of responsible AI (plus recommendations for research, science policy, and broader societal issues).^83^ These were formulated during a workshop with AI researchers, organized by the Future of Life Institute, a Boston-based non-profit research organization. Furthermore, the ‘Montreal Declaration for a Responsible Development of AI’ promulgates 10 principles which also partly coincide with the Table 2 principles for responsible AI.^84^ The outcome of a forum at the university of Montreal in 2017, involving hundreds of participants, the declaration ‘incorporates the concerns of all stakeholders in the field’^85^ and is intended to ‘spark a broad dialogue between the public, the experts and government decision-makers’.^86^ Finally, the UNI Global Union, a global federation of trade unions, has formulated the ‘Top 10 principles for ethical AI’, which at least partly cover the responsible AI principles (as in Table 2).^87^ These principles are to be used by trade unions on the shop floor as demands for responsible AI.

As far as civil society is concerned, the 24 companies on my list have as a rule developed links focussing on principled AI with a whole spectrum of activist, professional, and academic organizations (through workshops, conferences, and the like); they talk with them incessantly and regularly report about these conversations on their websites. The question is: did the pressures from civil society (as prominently expressed in such declarations about responsible AI) result in any accommodating steps by the committed firms (listed in Table 1)? Did they accept a standard, a seal, a certification scheme (like for non-biased AI) or take specific action (like freezing the sale of emotion AI)—either in harmonious cooperation with civil society forces (private co-regulation), or as the outcome of being pressured by them (civil regulation)? It turns out that such regulatory outcomes have not been forthcoming—no material of the kind has been found. Regulation by civil society in one form or another has not materialized.^88^

Even the modest beginning of just subscribing to declarations by civil society actors is (mostly) one bridge too far for them as I will show. Let me return to the calls for responsible AI promulgated by civil society. As far as these can be signed at all (the one from the UNI Global Union cannot), industrial signatures turn out to be far and few between. While the Universal Guidelines for AI are not signed by any firm and the Toronto Declaration has two company signatures, the Montreal Declaration has at any rate been signed by dozens of smaller, mostly Canadian, AI companies and consultancies. However, AI companies from my list of committed companies are *not* amongst the signatories.^89^ The Asilomar Principles, finally, have garnered the signatures of hundreds of robotics researchers (most of them having contributed to their drafting in the first place). Dozens of them work for companies that figure on my list of committed companies (Table 1). For example, researchers from IBM (five in toto) and Google (three in toto) are well represented. Their signatures, however, commit them as *individuals* only—the organizations they belong to are not bound by their signatures.

So, the 24 committed companies that I have been investigating just do not want to accommodate civil society actors concerning responsible AI—let alone yield to pressure from them. The forces of civil society—their lobbying efforts obviously not strong enough to impose themselves—are kept at a distance.^90^ However, at the time of finishing this manuscript, one prominent exception to this rule occurred: the case of facial recognition software. Mid-2020 Amazon and Microsoft announced that they would pause its sale to police departments, while IBM even decided to stop selling and researching the software altogether. However, these decisions were *not* so much inspired by the organizations of civil society and their declarations mentioned above. Instead, they were mainly prompted by continuous societal pressure over the years from organizations like the American Civil Liberties Union (ACLU), supported by individual AI researchers from both academia and firms selling facial recognition software. This appears to be the first instance of civil regulation proper of AI materializing.^91^

### Governmental Regulation of AI

It has to be borne in mind that a great many governmental organizations, at both national and international levels, have also been pushing for principled AI from 2016 onwards. Their statements about proper principles for AI are not to be mistaken.^92^ Ultimately, these recommendations may converge on new policies and fresh legislative proposals. What about the attitude of the 24 committed companies towards such regulation by the state? Table 4 gives an overview of the results. As can be seen, just 10 of them have issued statements about governmental regulation of AI. These have their headquarters either in Europe or in the USA, each with their own distinct regulatory ‘climate’; or they are truly global (McKinsey).

#### European Companies

Let me start with the eight committed AI companies from Europe on my list: just three of them have taken an explicit stance on the matter (Telefónica, Tieto, and SAP). European preparations for regulation of AI, driven by the European Commission, have been going on for years now (cf. their recent ‘White Paper on AI: a European approach to excellence and trust’^93^ that focusses on *ex ante* regulation of high-risk AI). For Telefónica and Tieto, in the meantime, this governmental regulation has become a fact of life. Acquiescing in the approach, they take part in the many EU deliberations about it and just try to soften the bureaucratic edges that—in their view—might hinder innovation.

Their largely concurring statements can be paraphrased as follows.^94^ The definition of high-risk AI activities is to be further specified (with high opportunity also to be considered in the definition), the provision of concrete use cases of high-risk AI would help to reduce uncertainty for the companies involved, and the focus is to be on specific AI applications (such as autonomous vehicles)—not on AI technologies. More importantly, regulation of AI risks being slow and hindering innovation. It therefore has to become ‘smarter’: experiment with so-called ‘regulatory sandboxes’ in which companies can try out innovative approaches while the regulator keeps close watch and may intervene if necessary.^95^ Telefónica summarizes this approach nicely: ‘Industry self-regulation, policy modernization and smarter regulatory supervision should be combined for a new approach’.^96^ Note that this is nothing other than a plea for modernized ‘enforced self-regulation’.^97^

As for SAP, earlier on (in 2018), the German business software company had tried to stem the tide by adopting the position that no new specific EU regulation on AI would be needed: ‘The current EU regulatory framework is sufficiently robust and does apply to AI. We caution policy makers against rushing into specific AI legislative initiatives that could hinder the development of AI and create legal inconsistencies’.^98^ The only action that they recommended was the review of existing legal frameworks as to their applicability towards issues of AI—in particular, privacy, consumer protection, liability, and intellectual property rights. Subsequently, the EU Commission has effectively brushed their position aside by insisting on new ethical guidelines and (ultimately) legislation for AI—and SAP has proceeded to join the deliberations on them (by becoming a member of the AI HLEG and engaging in advocacy lobbying concerning European legislation).

#### US Companies

Let us next turn to the 11 committed companies on my list headquartered in the USA. Table 4 shows that six of them have issued elaborate statements about governmental regulation of AI—all of them *in favour* of such regulation; or rather, more accurately, with each of them emphasizing the need for particular AI technologies or particular AI applications to be regulated.^99^ This is remarkable, since until recently they largely kept silent about regulation—self-regulation being their default position. But from 2018 onwards, their stances have evolved. Microsoft was the first large company to publicly embrace governmental regulation concerning AI; soon after, other large companies reluctantly followed suit. Their changing attitudes towards regulation will be presented below in chronological order of their ‘conversion’. Moreover, I treat Microsoft, Amazon, IBM, and Google first, since their conversions were mostly triggered by the facial recognition issue; subsequently, I present the positions adopted by Intel and Facebook, which are mainly related to the issue of privacy.

Microsoft has arguably been the first large company (involved in AI) to argue for new AI legislation. In January 2018, in a book about the future of AI, they still held a cautious position which can be summarized as follows.^100^ Current laws do already, to some extent, protect privacy and security of personal information, govern credit or employment decisions, and the like. ‘AI law’ will inevitably emerge as a new legal topic, but ‘before devising new regulations or laws, there needs to be some clarity about the fundamental issues and principles that must be addressed’.^101^ Stakeholders need sufficient time to identify the key principles for AI and implement them by adopting best practices.

Half a year later, the company drastically changed course: the time for deliberation was over. Spurred by the bitter controversies over facial recognition software (on account of the threat of ubiquitous surveillance and charges of gender and racial bias), Microsoft published a blog that argued that ‘the only *effective* way to manage the use of technology by a government is for the government proactively to manage this use itself. […] This in fact is what we believe is needed today — a government initiative to regulate the proper use of facial recognition technology […]’. Bi-partisan expert committees of Congress should prepare future legislation for its use in the USA. Note that the blog did not fail to mention that Microsoft, inspired by the same arguments, had been in support of the regulation of privacy for the last 15 years (the GDPR in particular).^102^

Next, let me discuss Amazon, their Amazon Web Services (AWS) in particular. The company has long tried to remain aloof from discussions about ethical AI and take no position. In the words of AWS executive Peter Stanski: ‘It is up to clients to decide whether their use of AWS tools is ethical’.^103^ Several controversial AI-related issues forced the platform to change tack. First, their AI recruiting tool had to be halted (2017), since it exhibited bias towards non-whites and women. Subsequently, their facial recognition tool (Rekognition) came under attack. Leading AI researchers from industry and academia condemned the tool for gender and racial bias, and their own employees and shareholders urged the company to halt the sale of the tool to law enforcement agencies. Ultimately, after two years of haggling, Amazon reluctantly suspended the sale to police departments for a year (June 2020).^104^ In the process, AWS proposed guidelines for future legislative regulation of facial recognition software in law enforcement (human review of results, a confidence level of 99%, transparency reports, public notification whenever video surveillance and facial recognition are in combined use) (February 2019).^105^ Thus, for the first time, AWS gave their support to regulation of a specific AI application. Jeff Bezos formulated his pragmatic approval as follows: ‘It’s a perfect example of something that has really positive uses, so you don’t want to put the brakes on it. At the same time, there’s lots of potential for abuses of that technology, so you do want regulation’.^106^

IBM has also been drawn into the regulation debate because of facial recognition issues. In 2019, they instituted their IBM Policy Lab that is tasked with developing propositions for policies for the digital age. Their approach towards governmental regulation of AI is designated as ‘Precision Regulation’. Such regulation is to target *specific* applications of AI and analyse in detail where along the chain of application risks to society may occur. Subsequently, regulatory rules have to be formulated *in proportion* to the risks to be contained: more stringent rules for high-risk situations, more relaxed rules for low-risk situations. In particular, they propose that governments designate standard developing organizations of choice (like NIST and CENELEC) and ask them to develop international standards. Adherence to these standards (as exemplified in certification) would be evidence of compliance with the law (‘safe harbour protection’).^107^ As a novel element, the corporation proposes that governments finance AI test beds in which stakeholders from civil society can put forward their point of view about responsible AI.^108^ In further notes about respectively harmful content online and facial recognition technology, IBM explicitly presents this approach as allowing to steer between the extremes of laissez-faire on the one hand and a blanket ban on the other.^109^ Clearly, IBM is a protagonist of states and markets jointly establishing a regulatory regime—the tenor is one of hybrid regulation.^110^

Google was the last company to join the chorus of firms clamouring for fresh regulation. Throughout 2019, it had stuck to the position that ‘in the majority of cases, general legal frameworks and existing sector-specific processes will continue to provide an appropriate governance structure’.^111^ Acknowledging that sometimes ‘additional oversight’ might be needed, their report about AI governance declared that ‘we look forward to engaging with governments, industry practitioners, and civil society on these topics’—for example, topics related to new weapons or police surveillance.^112^ Then, in February 2020, their CEO Sundar Pichai published an article in the *Financial Times*, in which he came around to AI regulation: ‘[…] there is no question in my mind that artificial intelligence needs to be regulated’. Governments have to assume a regulatory role. For regulation to be sensible, it must ‘take a proportionate approach, balancing potential harms, especially in high-risk areas, with social opportunities’. While sometimes existing frameworks suffice (such as the GDPR for privacy, and regulation of medical devices), new frameworks may also be needed, as for self-driving vehicles.^113^ In a Brussels’ interview (20 February 2020), he even lent support to a temporary ban on facial recognition technology.^114^

The last two US firms to be discussed, Intel and Facebook, are mainly concerned with AI regulation from the perspective of privacy. Their positions have also been shifting gradually. In 2017, Intel just stated that regulators should exercise oversight and intervene where necessary—but within the existing legal frameworks. Companies were to be guided by the Fair Information Practice Principles (as formulated by the OECD) and be able to demonstrate to regulators that they adhere to them.^115^ One year later, Intel slightly changed its tune and pleaded for ‘new legislative and regulatory initiatives’ (October 2018). These should be comprehensive, meaning that all potential issues of privacy are to be covered by these new laws. Moreover, these should be technology neutral. At the same time, they should support the free flow of data.^116^

Facebook, on their part, has also long been averse to any regulation of their activities. However, as pressures on both sides of the Atlantic mounted on account of data leaks and apparent privacy violations, the firm realized that this attitude would harm their interests in the long run. CEO Mark Zuckerberg therefore changed course and declared that issues such as harmful content and hate speech, election integrity, privacy, and data portability were areas in which new regulation would be welcome (30 March 2019).^117^

Let me confine myself to their position on two of those issues that are becoming more and more dependent on AI: privacy and harmful content. As concerns privacy, Zuckerberg has publicly embraced the GDPR, both as actually in force in Europe and as a model to be pursued in the US legislation.^118^ It remains to be seen, of course, whether Facebook’s actual data practices from then on have respected the letter (let alone the spirit) of the European Directive. Concerning harmful content, Facebook has seized the initiative and published a detailed report about possible ways to regulate such content, distinguishing between the approaches of ‘procedural accountability’ and of meeting ‘performance standards’ for taking down harmful content.^119^ Currently, they actually police incoming content themselves along these lines. By their own estimate, hate speech and misinformation detected on their platform are mostly (up to 90%) discovered by AI tools—either based on natural language modelling or capable of identifying multimodal hate speech (text plus images).^120^ The company has also initiated the establishment of an independent Oversight Board, which is to decide about individual cases brought forward and about content policies.^121^ This board represents a form of self-regulation, instituted in lieu of (inter)national regulation which is, of course, a politically charged topic that is unlikely to be successful in the short run. Finally, during the 2020 US elections and their aftermath, Facebook (Facebook, WhatsApp) —as well as Google (YouTube) and Twitter—came under heavy fire and initiated their own stricter censoring of hateful rhetoric and disinformation on their social media—mainly by relying more heavily on automated censoring with AI tools.^122^

#### Global Companies

Finally, it remains to present the position of McKinsey & Co on regulation of AI. This consulting firm no longer maintains a corporate headquarters—thereby straddling the regulatory regimes of the EU and the USA, of the whole world actually. It has published detailed reports about the future of AI in healthcare, education, and the automotive sector.^123^ Throughout them, it stresses the need for companies to proactively engage with regulators and regulatory issues—to them, there is simply no way around it. Suggestions for the regulatory regime (particularly in healthcare)^124^ are manifold: standards are to be created proactively together with governments, regulatory sandboxes are to be instituted, governments are to establish centres of excellence (populated by experts on issues of AI) that support the creation of regulatory rules concerning specific AI applications, and frameworks for accountability and liability concerning AI are to be determined. In these remarks, we observe again (as with Telefónica and Tieto) a plea for ‘smarter’ hybrid regulation.

## Regulation of AI: Overview and Discussion

The remarks about self-regulation at the industry level made by the 24 AI companies committed to AI principles predominantly refer to the need for future best practices and standards. As far as regulation by or co-regulation with civil society is concerned, a phenomenon well known in the case of environmental issues, no evidence has been found that civil society actors have effectively exerted any regulatory influence concerning AI on the corporations on my list of 24 (or any other firms for that matter); the committed companies succeeded in keeping the pressure from civil society at bay. Only recently (mid-2020), an exception came to the fore: Amazon and Microsoft were effectively forced to halt the sale of facial recognition software to police departments—IBM even decided to abandon that line of software altogether. Concerning governmental regulation of AI, though, many lengthy comments were aired, all pointing to a definite need for such regulation. These explicit ‘conversions’ to the view that AI needs to be regulated, surprising as they are, deserve some more analysis.

The statements—by three European companies, six US companies, and one global company on my list—exhibit some remarkable commonalities. As a model for regulation of AI, the *risk-based approach* has widely been embraced: the higher the risks that a specific AI application in a specific context of use generates, the tighter the regulation required. In Europe, the approach has already become the norm for law makers (and as such at the heart of the EU ‘White Paper on AI’)^125^; the European companies in my sample have come to take it for granted. In the USA, the approach has only more recently gathered public attention. Especially, IBM and Google constitute a kind of vanguard with their explicit pleas to follow the European example and embrace risk-based regulation wherever applicable.

Noticeably, several comments from the companies involved indicate efforts towards softening the edges of ‘hard’ regulation. IBM recommends that governments designate bodies of choice that develop standardization and certification processes that guarantee conformity with the law, while both Telefónica and Tieto (as well as McKinsey) toy with the idea of regulatory sandboxes that give participants free rein to experiment with AI—both examples of hybrid regulation with the state receding into the background.

Further, some of the above comments explicitly place such regulation on a regulatory scale: between laissez-faire on the one hand and a ban or moratorium on technology uses on the other. Risk-based regulation gets imbued with the idea of being the ‘reasonable’ *middle course*: avoiding societal catastrophes while letting innovation and economic activity continue. Let me quote from comments by Microsoft and IBM. As concerns facial recognition technology, Microsoft emphasizes that ‘unless we act, we risk waking up five years from now to find that facial recognition services have spread in ways that exacerbate societal issues’. A ‘commercial race to the bottom, with tech companies forced to choose between social responsibility and market success’, is to be avoided. Instead, a ‘floor of responsibility’ is to be built, and only the rule of law can do so.^126^ Note that Microsoft has always been vehemently opposed to bans of any kind. In a similar vein, IBM stresses that their ‘precision regulation’ represents the reasonable balance par excellence: it ‘emphasizes carefully targeting policy to address legitimate concerns while promoting innovation […]’. About possible bans, Big Blue remarks that ‘[…] blanket bans on technology are not the answer to concerns around specific use cases. Casting such a wide regulatory net runs the very real risk of cutting us off from the many — and potentially life-saving — benefits these technologies offer’.^127^

In the above statements by the US companies about regulation of AI, many aspects of applied AI are mentioned as involving risks and therefore suitable candidates for regulation: issues such as privacy and online content moderation, and applications such as biometrical identification, self-driving cars, and drones. But no application has drawn so much attention, nay, ire, as facial recognition software. It has been a real *catalyst* for fuelling discussions about the future regulation of AI. The risks involved are those of biased results (against non-whites and women in particular), privacy violations, and unrestrained mass surveillance. Over the last four years, several incidents have been reported of such software malfunctioning. At various times Google, Amazon, Microsoft, and IBM have all been the target of serious criticisms of the kind, mainly voiced by AI researchers from various corporations and academia and by the ACLU. As a result, their software offerings had to be updated repeatedly. Ultimately, amidst the Black Lives Matter protests, Amazon and Microsoft decided to pause their sales of facial recognition services to the police. IBM even decided to leave the facial recognition business altogether.

Simultaneously, a flurry of new US laws have been proposed at both federal and state levels. These target not only facial recognition software but also more broadly biometrical identification, privacy, algorithmic decision-making, and more. Restrictions are often tailored to the specific use context: companies, public spaces, public institutions, police departments, shops, state rental units, etc. A focus on actually getting new regulatory legislation accepted has, we may conclude, actually been set in motion.^128^

In these deliberations, for one thing, many civil society organizations take part. Especially, the ACLU is a vocal critic of new technologies that appear to threaten civil liberties. Their position on facial recognition software has consistently been that it has to be put to a legal halt until stringent conditions have been met. The ACLU represents a formidable civil force inside these legislative debates.^129^ For another, the companies actually developing the new technologies at issue also take part in these deliberations on future laws. The committees involved as a rule invite all interested parties to show up and voice their opinions. Take the deliberations around the Washington State bill about facial recognition software. The main players took part: Amazon, trying to ease restrictions,^130^ as well as Microsoft with its agenda, which included trying to take a ban from the table.

This raises, of course, the shadow of *regulatory capture*. One such instance already appears to have occurred. Microsoft has been publishing repeatedly about facial recognition software. In December 2018, in particular, a Microsoft blog enumerated the issues that regulation of the software should address. As concerns possible bias, the law is to require full documentation, allow independent testing, make human review of consequential decisions based on facial recognition mandatory, and introduce guarantees against unlawful discrimination. If facial recognition is employed, considerations of privacy require putting up conspicuous notices and asking for consumer consent. Finally, in order to safeguard human rights, surveillance is only to be allowed in special circumstances.^131^ Remarkably, requirements of the kind have actually—after much haggling—ended up in the Washington State law that finally passed in March 2020 (Engrossed Substitute Senate Bill 6280).^132^ Not much of a surprise, since the bill had actually been written by a senator who is also employed by Microsoft (Joe Nguyen).

Noticeably, the same charge of regulatory capture can be heard in Europe. It has in particular been levelled against the AI HLEG set up by the EU. Corporate interests are argued to have a disproportionate influence on this advisory body (Opoku, 2019, Vasse’i 2019). Reportedly, these have prevented the mentioning of bans (non-negotiable ‘red lines’) on lethal autonomous weapons and social credit scoring systems in the EU ‘Ethics Guidelines for Trustworthy AI’ (2019).^133^

A more thorough evaluation of the new approaches to regulation of AI and their actual implementation is indicated of course: what are the pros and cons of these regulatory efforts? What do they bring for society in general and for companies in particular? What role do the companies involved actually play in their shaping? Do their actions square with their stated intentions (as explored in Sect. 5)? To answer these questions, the time is not ripe though: (self-)regulation of AI is just starting to crystallize. The ultimate shape of standards, codes, laws, and their implementation will be what matters. Only if we are able to zoom in on all their details will judgments be possible. Anyway, whenever the time is right, it will be an undertaking of its own, going far beyond my present framework of investigating AI companies and their pledges to the cause of responsible AI.

## Conclusion: Towards Proper Accounting for Responsible AI

From 2016 onwards, as many as 24 AI companies worldwide have publicly committed to principles for responsible AI (Table 1). Between them, the principles are virtually identical (Table 2). Subsequently some of these firms (cf. Table 3) instituted appropriate governance mechanisms internally, provided their employees with new materials and training about principled AI, developed novel software tools for each of the core qualities of such AI (fairness, explainability, security/privacy), and drafted proposals for accountability. Also, a couple of these firms are currently devoting large funds to newly erected academic departments or programmes focussing on research into aspects of responsible AI. Although the ‘new governance’ for responsible AI needs a more thorough appraisal, all other efforts, in my view, definitely represent steps forward on the road to responsible AI. Charges that the community of AI experts has a ‘preoccupation with narrow computational puzzles’ which betrays a penchant for ‘technological solutionism’ may be accurate, but the fruits of those preoccupations and penchants constitute indispensable elements for making progress towards responsible AI.

Further, several European and American companies on the list of committed firms (Table 1) have publicly declared that fresh state regulation of AI is the preferred option for the future (cf. Table 4). Especially for the US companies amongst them, this represents a drastic break with past convictions, probably having much to do with the continuous societal unrest about the uses of facial recognition software. The firms involved appear to bet on reining in the high-risk applications of AI, as a middle road between outright banning technology and laissez-faire. This willingness to let their AI applications be regulated by society may also, in my view, be considered a step forward—although the danger of regulatory capture looms large in view of the fact that the companies in question not only have large amounts of money at their disposal for lobbying activities, but also command a large proportion of the AI capabilities in society—which they will not hesitate to put to proper use in the regulatory process.

What has to happen for the responsible AI agenda to be pushed forward? Obviously, the agenda has to be embraced by larger segments of society, in particular by the great many other AI companies that are not to be found on my list. The 24 committed AI companies, though, especially those that put the AI principles into practice and embraced high-risk regulation, can also continue to contribute. As a vanguard, they may try and *connect* the two strands presented above. Their members have promised to deliver only AI that is responsible and declared that they are willing to let themselves be subjected to appropriate legislation. Let them take one further step and affirm that they will accept any invitation to actually *be called to account* by regulatory agencies in sectors such as finance, health, justice, education, and the like. Instances of AI companies actually complying with requests for accountability in a spirit of cooperation would further the cause of principled AI.

Studies about accountability concerning AI are steadily accumulating and can be of use in the process. Compare a report from AI Now (2018) that argues that existing regulatory frameworks for the US public agencies (in sectors such as health and criminal justice) have to be supplemented with fresh practical frameworks to assess automated decision systems.^134^ The agencies are to be enabled to perform ‘algorithmic impact assessments’ (focussing on aspects such as bias and fairness in AI) which may include ‘external researchers and auditors’ in the process. Assessments are to take place before an AI solution is reached as well as regularly afterwards, while the system is running. Due process mechanisms are to be made available to affected communities.

A report from the Partnership on AI (2019) represents an even more radical proposal.^135^ Focussing on the use of AI risk assessment tools in the criminal justice system, it argues that an independent outside body consisting of ‘legal, technical, and statistical experts, currently and formerly incarcerated individuals, public defenders, public prosecutors, judges, and civil rights organizations’ must regularly perform audits of the tools (with a focus on all aspects of responsible AI).^136^ In particular, ‘training datasets, architectures, algorithms, and models’ should be available to outside research communities for criticism.^137^

This final step of AI companies actually conforming to public audits and readily providing all necessary details about their algorithms is a spectacle that has to unfold yet. Fortunately, Google, Microsoft, and IBM themselves have developed novel accountability tools (cf. above) that may be put to good use when assessments as just mentioned have to be performed. However, scepticism whether this will happen any time soon is not unwarranted, since the argument that algorithmic details need to be protected as trade secrets still seems to enjoy support amongst AI companies both large and small. As a result, any ‘data sheets’, ‘model cards’, or ‘fact sheets’ submitted risk to be full of unfilled blanks, while companies prefer to leave out ‘secret’ information.

## Appendix: On Search Method

My aims in this research were the following:

1. To identify companies practicing and developing AI in order to apply/sell/advise about AI (‘AI companies’) that have recently publicly committed to AI principles or AI guidelines;
2. To investigate whether they have introduced internal governance tools for responsible AI (an ethics committee, an advisory board, guidelines, courses, checklists, training, new software tools, etc.);
3. To investigate whether they have published proposals about proper governance of AI (best practices, standards, ethical codes, regulation, audit, etc.).

In the search process to be explained below, I used the following three series of keywords in Google search and in some other search engines (with cookies and history deleted):

- *Keywords series 1 (KS1):* “responsible AI” OR “ethical AI” OR “trustworthy AI” OR “AI principles” OR “AI guidelines”. Further, in searches, the keywords mentioned have been amalgamated into several suitable combinations (such as “ethical AI principles”), and AI has been replaced by “artificial intelligence”;
- *Keywords series 2 (KS2)*: the adjectives advisory/ethical/ethics have variously been combined with the nouns board/committee/council/panel/team, to form actual keywords such as “advisory council” or “ethical team”. Further search with guidelines OR training OR tool(s) OR algorithm OR bias OR fair(ness) OR explainable/explainability OR interpretable/interpretability OR robust(ness) OR secure/security OR privacy OR accountable/accountability;
- *Keywords series 3 (KS3)*: “best practice(s)” OR standard(s) OR “ethical code(s)” OR regulation OR governance.

While searching with a series of keywords, these were usually not all included in one search only but spread out in portions over several searches.^138^

### Ad 1: AI Companies Committed to AI Principles

As explained in the main text, I first consulted several sources to identify ‘AI companies’ committed to AI principles. Subsequently, in order to update the 18 results obtained, I performed a limited supplementary Google search of my own, with the same keywords as Jobin et al. (2019) plus some more (KS1). The first 30 results were checked; results that identified committed AI companies not yet known to me were added to the list. This yielded just one more result.

Secondly (as also explained in the main body), the list of for-profit partners of the PAI has been inspected for identification of new candidates for my list: five new company names emerged. Subsequently, I staged a Google search for each of them in order to establish whether they have also *explicitly* committed to AI principles (so had escaped my attention thus far). Taking Affectiva as an example (from now on), the company’s website affectiva.com was searched manually with words from KS1. Each time, the first 30 search results were inspected. The outcome of this exercise confirmed that, indeed, none of the five added PAI members had explicitly subscribed to AI principles of any kind.

### Ad 2: Internal Governance Tools for Responsible AI

After the compilation of this list of ‘committed’ AI companies (24 in total; Table 1), each of them was searched for documents mentioning keywords from series 2. First, a company’s website (such as affectiva.com) was searched with words from KS2, always combined with the term AI. Secondly, a site-specific Google query was performed: [site: affectiva.com AND AI AND KS2]. Thirdly, a more general Google search was carried out starting with the company’s name: [Affectiva AND AI AND KS2]. Finally, the same search terms were employed within three quality journals (the *Financial Times*, *The New York Times*, and *The Washington Post*), using the journals’ internal search engines. Each time, the first 30 search results were inspected.

### Ad 3: Governance of AI

The ‘committed’ companies were finally searched for documents mentioning keywords from series 3. As before, I carried out three consecutive searches: a manual search of a company’s website using AI AND KS3; the site specific Google query [site: affectiva.com AND AI AND KS3]; and the more general search [Affectiva AND AI AND KS3], both on Google and on the three quality journals’ websites. Each time, the first 30 search results were inspected.

The outcomes of searching with KS2 and KS3 are listed in condensed form in Tables 3 and 4. The contents of the documents thus identified are discussed more fully in the article.

## Footnotes

1 Floridi (2019: 188) proposes the term ‘digital ethics lobbying’ for this practice, defined as ‘exploiting digital ethics to delay, revise, replace, or avoid’ appropriate legislation of digital matters.

2 AI HLEG (2019) A Definition of AI: Main Capabilities and Disciplines. Definition Developed for the Purpose of the AI HLEG’s Deliverables. Available at https://ec.europa.eu/digital-single-market/en/news/definition-artificial-intelligence-main-capabilities-and-scientific-disciplines.

3 https://alanwinfield.blogspot.com/2019/04/an-updated-round-up-of-ethical.html.

4 https://cyber.harvard.edu/publication/2020/principled-ai.

5 https://futureoflife.org/ai-policy/.

6 https://hai.stanford.edu/research/ai-index-2019: 272–274.

7 https://inventory.algorithmwatch.org/.

8 https://www.cs.ox.ac.uk/efai/resources/alphabetical-list-of-resources/.

9 https://www.nesta.org.uk/data-visualisation-and-interactive/ai-governance-database/.

10 Quoted from https://www.partnershiponai.org/membership/. For the precise content of those tenets, see Table 2.

11 Statistics from https://ec.europa.eu/transparency/regexpert/index.cfm?do=groupDetail.groupDetail&groupID=3591&NewSearch=1&NewSearch=1.

12 Note the contrast with the PAI: organizational commitment to AI principles is a condition for membership.

13 AI HLEG (2019) Ethics Guidelines for Trustworthy AI: p. 1. Available at https://ec.europa.eu/digital-single-market/en/news/ethics-guidelines-trustworthy-ai.

14 As always with classification, there are borderline cases to decide upon. What is to count as a commitment to AI principles, what is to count as a substantial involvement in AI development? An example is Deloitte, a global management consultancy, which has, in a fashion, committed to AI principles (‘Digital Ethics proposition’) and developed a responsible AI tool (their open sourced ‘GlassBox’). Since these are just first steps, I consider them to be not (yet) committed and substantially involved in (responsible) AI and have therefore not put the firm on my list of committed AI companies.

15 Noticeably, Jobin et al. (2019) studied available declarations of AI principles (for *all* kinds of organizations involved) and tabulated the empirical frequencies of AI principles amongst them (Jobin et al., 2019: their Table 3). The associated ordering of the headings (for the various AI principles) is almost identical to the one I obtained (my Table 2).

16 AI HLEG (2019) Ethics Guidelines for Trustworthy AI: p. 14 ff. Available at https://ec.europa.eu/digital-single-market/en/news/ethics-guidelines-trustworthy-ai.

17 Darrell M. West (2018) The role of corporations in addressing AI’s ethical dilemmas. Brookings Institution. September 13, 2018. Available at https://www.brookings.edu/research/how-to-address-ai-ethical-dilemmas/.

18 R. Sandler, J. Basl (2019) Building data and AI ethics committees. Accenture. Available at https://www.accenture.com/_acnmedia/pdf-107/accenture-ai-and-data-ethics-committee-report-11.pdf.

19 In their report, Sandler and Basl elaborate on the building of such an ethical committee: its composition, powers, and position in the organization, and the review procedures it may adopt.

20 Darrell M. West (2018) Brookings survey finds divided views on artificial intelligence for warfare, but support rises if adversaries are developing it. Brookings Institution website, August 29, 2018. Available at https://www.brookings.edu/blog/techtank/2018/08/29/brookings-survey-finds-divided-views-on-artificial-intelligence-for-warfare-but-support-rises-if-adversaries-are-developing-it/.

21 Singapore Digital, IMDA (Infocomm Media Development Authority), and PDPC (Personal Data Protection Commission) (2020) Model Artificial Intelligence Governance Framework, second edition. Available at https://www.pdpc.gov.sg/-/media/Files/PDPC/PDF-Files/Resource-for-Organisation/AI/SGModelAIGovFramework2.pdf.

22 Consultancies Accenture and McKinsey are included in this number, but actually constitute a special case. They *do* advise clients to adopt responsible AI governance and practices but have no need for such governance inside their *own* firm.

23 It should be noted that SAP and Salesforce also took measures to increase the diversity of their teams.

24 Although DeepMind is a borderline case, it only installed ‘external fellows’ on an individual basis, not as a proper group.

25 Google proposed an external council but had to cancel it soon after because employees protested against some of the proposed members: cf. https://blog.google/technology/ai/external-advisory-council-help-advance-responsible-development-ai/.

26 For both quotes, see https://news.sap.com/2018/09/sap-guiding-principles-for-artificial-intelligence/.

27 Which is not always the case: names of council members at Salesforce are unknown.

28 https://www.microsoft.com/en-us/ai/our-approach?activetab=pivot1%3aprimaryr5.

29 The CausalNex tool developed by McKinsey as mentioned in Table 3 is just a tiny contribution which is disregarded here.

30 Facebook open sourced one of these tools (Captum).

31 Here, I disregard their internal blogposts about responsible AI (as mentioned in Table 3) since these can at most be interpreted as the tiny beginnings of training options for their personnel.

32 At https://trailhead.salesforce.com/content/learn/modules/responsible-creation-of-artificial-intelligence.

33 At https://www.salesforce.com/blog/2019/04/einstein-discovery-ai-outside-salesforce.html.

34 https://pair-code.github.io/facets/

35 See https://pair-code.github.io/what-if-tool/.

36 For more details about fairness measures cf. http://aequitas.dssg.io/static/images/metrictree.png.

37 See materials at https://fairlearn.github.io/contents.html, and https://github.com/fairlearn/fairlearn.

38 This paragraph is based on https://aif360.mybluemix.net/.

39 For an overview of possible techniques for explanation of AI relevant to organizations using AI see ICO/Alan Turing Institute (2019). Explaining decisions made with AI: especially annexes 2 and 3. Available at https://ico.org.uk/for-organisations/guide-to-data-protection/key-data-protection-themes/explaining-decisions-made-with-artificial-intelligence/. A more mathematical overview is provided by Molnar (2020). Interpretable Machine Learning; A Guide for Making Black Box Models Explainable. Available at https://christophm.github.io/interpretable-ml-book/.

40 See respectively https://ai.google/responsibilities/responsible-ai-practices/?category=interpretability; https://www.ibm.com/blogs/research/2019/08/ai-explainability-360/; https://docs.microsoft.com/nl-nl/azure/machine-learning/how-to-machine-learning-interpretability and https://github.com/interpretml/interpret, https://github.com/interpretml/interpret-community.

41 The sequel is based on https://pair-code.github.io/what-if-tool/learn/tutorials/walkthrough/.

42 More details on https://aix360.mybluemix.net/ and https://aix360.mybluemix.net/resources#guidance.

43 The sequel is based on https://docs.microsoft.com/nl-nl/azure/machine-learning/how-to-machine-learning-interpretability.

44 The LIME option on the Microsoft website seems to be discontinued at the time of finishing this manuscript.

45 For more details, see the useful classification developed by Microsoft in: Failure modes in Machine Learning. Available at https://docs.microsoft.com/en-us/security/engineering/failure-modes-in-machine-learning#. I present almost all of the ‘intentionally-motivated failures’ mentioned in this document and sort them along the lines of confidentiality and system integrity.

46 https://ai.googleblog.com/2017/04/federated-learning-collaborative.html.

47 https://developer.ibm.com/open/projects/adversarial-robustness-toolbox/.

48 https://docs.microsoft.com/en-us/azure/machine-learning/concept-differential-privacy; https://github.com/opendifferentialprivacy/whitenoise-core.

49 https://github.com/tensorflow/cleverhans.

50 https://github.com/tensorflow/privacy/blob/master/tutorials/walkthrough/README.md.

51 http://www.cleverhans.io/privacy/2018/04/29/privacy-and-machine-learning.html.

52 https://fuchsia.googlesource.com/cobalt.

53 https://www.ibm.com/blogs/research/2019/11/ibm-joins-stanford-partner-program/?linkId=77045217.

54 https://about.fb.com/news/2019/01/tum-institute-for-ethics-in-ai/.

55 https://developer.amazon.com/blogs/alexa/post/d9a995d4-943a-456b-9527-848f437b1a8c/amazon-and-nsf-collaborate-to-accelerate-fairness-in-ai-research; https://www.amazon.science/3-questions-about-the-amazon-national-science-foundation-collaboration-on-fairness-in-ai.

56 https://news.nd.edu/news/notre-dame-ibm-launch-tech-ethics-lab-to-tackle-the-ethical-implications-of-technology/.

57 Over the period 2016–2020, as a whole, Amazon and Facebook delivered just a few publications about each of the aspects of responsible AI discussed (fairness/bias, explainability/interpretability, security/privacy). Google, Microsoft, and IBM on the other hand each published many more papers on each of these aspects: at least more than ten, usually dozens of them (source: R&D reports on respective company websites). For security and privacy, the output numbers are highest.

58 Under the rubric of possible vehicles for ethics washing, Bietti (2020) explicitly mentions ethics councils, the hiring of moral philosophers, and the funding of efforts to develop fair ML, while Benkler (2019) mentions the failed Google ethics council and the R&D funds for responsible AI provided by Facebook (in collaboration with the TU Munich) and Amazon (together with the NSF). Note that Benkler introduces a new topic for debate besides the one about ethics washing: is it justified for universities to accept these funds, even without (seemingly) any strings attached? I leave that discussion aside.

59 Note though that no hard evidence of such hopes has surfaced in my research; it would require a more in-depth analysis. Only accidentally we obtain a glimpse of inner company resistance to regulation being real—such as when an internal Google memo leaked out that proposed a campaign against the EU and its Commission to influence forthcoming regulation about the rules for the Internet (November 2020).

60 See https://www.zdnet.com/article/google-employee-protests-now-google-backs-off-pentagon-drone-ai-project.

61 She co-authored the papers about ‘datasheets for datasets’ and ‘model cards’ as discussed in Sect. 3.2.4.

62 More details in Karen Hao. We read the paper that forced Timnit Gebru out of Google. Here’s what it says. *MIT Technology Review*, 4 December 2020.

63 She co-authored the paper about ‘model cards’ as discussed in Sect. 3.2.4.

64 More references about this issue of ‘technological fixes’ in Whittaker et al. (2018) and Crawford et al. (2019).

65 Note that there are also companies that have *not* committed to any principles for AI but have nevertheless introduced many of the tools in question. A case in point is H2O, which has even open sourced its AI platform.

66 The 30 respondents in their study mostly worked in organizations that are members of the PAI (both for-profit and non-profit).

67 Source: https://www.mckinsey.com/featured-insights/artificial-intelligence/global-ai-survey-ai-proves-its-worth-but-few-scale-impact; see exhibit 4.

68 Note that a similar, though less extensive, typology of regulation is presented by Roger Clarke (2019). He discusses, from the perspective of realizing the responsible use of AI, the pros and cons of applying various types of regulation to AI.

69 Hence, Black (1996) has coined the term ‘coerced self-regulation’ in case this shadow looms large.

70 Black (1996) employs the term ‘sanctioned self-regulation’.

71 Note that, as before, I omit Apple, Samsung, Deutsche Telekom, Sony, Kakao, Unity Technologies, and Affectiva from the analysis, since they did not publish anything about this issue.

72 https://www.partnershiponai.org/resources/.

73 Just one non-member of the PAI on my list of 24 also recommended best practices (Telefónica).

74 SAP (2018) European Prosperity Through Human-Centric Artificial Intelligence; The Intelligent Enterprise: p. 28. Available at https://www.sap.com/documents/2018/01/3e67a134-ee7c-0010-82c7-eda71af511fa.html.

75 Accenture (2018). Realising the economic and societal potential of responsible AI in Europe: p. 23. Available at https://www.accenture.com/_acnmedia/pdf-74/accenture-realising-economic-societal-potential-responsible-ai-europe.pdf.

76 Accenture (2016). Building digital trust: The role of data ethics in the digital age. Available at https://www.accenture.com/_acnmedia/PDF-22/Accenture-Data-Ethics-POV-WEB.pdf.

77 Accenture (2016). Universal principles of data ethics; 12 guidelines for developing ethics codes. Available at https://www.accenture.com/_acnmedia/pdf-24/accenture-universal-principles-data-ethics.pdf.

78 Accenture (2016), Universal principles (…), cit., p. 3.

79 SAP (2018) ibidem.

80 Cf. the visualization of organizations committed to AI principles by the Berkman Klein Center at Harvard: https://wilkins.law.harvard.edu/misc/PrincipledAI_FinalGraphic.jpg. Civil society organizations are rendered in yellow.

81 The Toronto Declaration (2018) Available at https://www.torontodeclaration.org/declaration-text/english/.

82 The Public Voice (2018) Universal Guidelines for Artificial Intelligence. Available at https://thepublicvoice.org/ai-universal-guidelines.

83 Asilomar AI Principles (2017) Available at https://futureoflife.org/ai-principles/.

84 Montreal Declaration for responsible AI development (2018) Available at both https://www.montrealdeclaration-responsibleai.com/the-declaration; and https://5dcfa4bd-f73a-4de5-94d8-c010ee777609.filesusr.com/ugd/ebc3a3_506ea08298cd4f8196635545a16b071d.pdf.

85 https://nouvelles.umontreal.ca/en/article/2018/12/04/developing-ai-in-a-responsible-way/.

86 https://nouvelles.umontreal.ca/en/article/2017/11/03/montreal-declaration-for-a-responsible-development-of-artificial-intelligence/.

87 UNI Global Union (no year) Top 10 principles for ethical artificial intelligence. Available at http://www.thefutureworldofwork.org/media/35420/uni_ethical_ai.pdf.

88 As observed above, the PAI has nominally turned into a private co-regulation arrangement. It hardly engages in efforts, though, to gain acceptance for their recommendations across industry. It operates more like a think-tank than as a proper regulatory body. Incidentally, it is precisely for this lack of regulatory bite that Access Now recently resigned from the PAI (October 2020; cf. https://www.accessnow.org/access-now-resignation-partnership-on-ai/).

89 With one exception; Element AI, being Canadian, endorsed the Toronto Declaration.

90 Note that amongst all civil society organizations issuing declarations or guidelines as mentioned above, only the UNI Global Union may possess the required force. For years, this federation has been concluding agreements with multinational firms all over the world. Compare the recent agreement with Telefónica (May 2020), which is to guarantee the right of their employees to return to work safely in these times of COVID-19.

91 I come back to this episode more fully in Sect. 6 below.

92 Cf. the visualization of organizations committed to AI principles by the Berkman Klein Center at Harvard: https://wilkins.law.harvard.edu/misc/PrincipledAI_FinalGraphic.jpg. Governmental organizations are rendered in green and brown.

93 Available at https://ec.europa.eu/info/publications/white-paper-artificial-intelligence-european-approach-excellence-and-trust_en.

94 TietoEVRY (no year) EU level AI regulation. Available at https://www.tietoevry.com/globalassets/tietoevry-eu-level-ai-regulation-position-paper-v.1.pdf; https://www.telefonica.com/en/web/public-policy/blog/article/-/blogs/regulating-ai-a-risk-based-approach-for-europe; Telefónica (2018) A Manifesto for a New Digital Deal. Available at https://www.telefonica.com/documents/341171/0/digital-manifesto/b27e86b0-8a34-09d5-b908-67e1623cbc45.

95 Such sandboxes have been introduced in the financial sector from 2015 onwards (in about 20 countries). In them, for example, new algorithmic schemes for fraud detection have been pioneered.

96 Telefónica (2018) A Manifesto (..), cit., p. 91.

97 Apart from these close parallels, the two companies have their specific complaints. Digital platforms should urgently be regulated (Telefónica); AI for biometric identification is not to be slowed down let alone be banned, and personal data should be shared on the basis of opt-out (Tieto).

98 SAP (2018) European prosperity through human-centric artificial intelligence: The intelligent enterprise: p. 23. Available at https://www.sap.com/documents/2018/01/3e67a134-ee7c-0010-82c7-eda71af511fa.html.

99 Note that I do not take into account occasional public comments from company executives about governmental regulation of AI: e.g., Tim Cook (Apple), Marc Benioff (Salesforce), or Rana el Kaliouby (Affectiva)—all in support of AI regulation.

100 Microsoft (2018) The future computed: Artificial Intelligence and its role in society: chapter 2. Available at https://news.microsoft.com/cloudforgood/_media/downloads/the-future-computed-english.pdf.

101 Microsoft (2018) The Future Computed (..), cit., p. 74.

102 Quotes and paraphrases from https://blogs.microsoft.com/on-the-issues/2018/07/13/facial-recognition-technology-the-need-for-public-regulation-and-corporate-responsibility/.

103 http://cdn.computerworld.com.au/article/661203/aws-ethical-about-ai-we-just-don-t-talk-about-it-say-apac-execs/.

104 https://blog.aboutamazon.com/policy/we-are-implementing-a-one-year-moratorium-on-police-use-of-rekognition. Note that Microsoft followed suit a few days later.

105 https://aws.amazon.com/blogs/machine-learning/some-thoughts-on-facial-recognition-legislation/.

106 https://www.geekwire.com/2019/jeff-bezos-facial-recognition-perfect-example-need-regulation-amazon-working/.

107 In the scheme developed by Senden et al. (2015), this particular hybrid would be classified as ‘conditioned self-regulation’.

108 Ryan Hagemann & Jean-Marc Leclerc (2020) Precision Regulation for Artificial Intelligence. Available at https://www.ibm.com/blogs/policy/ai-precision-regulation/.

109 https://www.ibm.com/blogs/policy/cda-230/; and Christina Montgomery & Ryan Hagemann (2019) “Precision Regulation” and Facial Recognition. Available at https://www.ibm.com/blogs/policy/facial-recognition/.

110 Like Amazon and Microsoft, IBM has also halted the sale of facial recognition software; R&D efforts related to it were put on hold as well (June 2020). Big Blue argues that a national [USA] dialogue about its use by law enforcement agencies has to be conducted first. Sceptics speculate that the company had actually fallen behind on facial recognition software, so the societal hassle about its uses provided a perfect excuse for halting it altogether, under the mantle of social responsibility (cf. https://www.techspot.com/news/85554-ibm-no-longer-make-general-purpose-facial-recognition.html).

111 https://www.blog.google/outreach-initiatives/public-policy/engaging-policy-stakeholders-issues-ai-governance/.

112 Google (2019) Perspectives on Issues in AI Governance. Available at https://ai.google/static/documents/perspectives-on-issues-in-ai-governance.pdf.

113 Sundar Pichai (2020) Why Google thinks we need to regulate Ai. *Financial Times*, January 20, 2020. Available at https://www.ft.com/content/3467659a-386d-11ea-ac3c-f68c10993b04.

114 https://www.ft.com/content/0e19e81c-3b98-11ea-a01a-bae547046735.

115 Intel (2017) Artificial Intelligence: The Public Policy Opportunity. Available at https://blogs.intel.com/policy/files/2017/10/Intel-Artificial-Intelligence-Public-Policy-White-Paper-2017.pdf.

116 Intel (2018) Intel’s AI Privacy Policy White Paper: in particular, pp. 5–6. Available at https://blogs.intel.com/policy/files/2018/10/Intels-AI-Privacy-Policy-White-Paper-2018.pdf.

117 Mark Zuckerberg (2019) The Internet needs new rules: Let’s start in these four areas. Available at https://www.washingtonpost.com/opinions/mark-zuckerberg-the-internet-needs-new-rules-lets-start-in-these-four-areas/2019/03/29/9e6f0504-521a-11e9-a3f7-78b7525a8d5f_story.html.

118 Zuckerberg (2019) Ibidem.

119 Facebook (2020) Charting a way forward: Online content regulation. Available at https://about.fb.com/wp-content/uploads/2020/02/Charting-A-Way-Forward_Online-Content-Regulation-White-Paper-1.pdf.

120 https://www.technologyreview.com/2020/05/12/1001633/ai-is-still-largely-baffled-by-covid-misinformation/.

121 https://www.oversightboard.com/.

122 Note that for Europe, the Digital Services Act (a legislative proposal for now) proposes new rules for the elimination of harmful content online.

123 McKinsey (2017) Artificial Intelligence: the next digital frontier? Available at https://www.mckinsey.com/~/media/mckinsey/industries/advanced%20electronics/our%20insights/how%20artificial%20intelligence%20can%20deliver%20real%20value%20to%20companies/mgi-artificial-intelligence-discussion-paper.ashx; McKinsey (2017) Smart moves required: the road towards artificial intelligence in mobility. Available at https://www.mckinsey.com/~/media/mckinsey/industries/automotive%20and%20assembly/our%20insights/the%20road%20to%20artificial%20intelligence%20in%20mobility%20smart%20moves%20required/smart-moves-required-the-road-towards-artificial-intelligence-in-mobility.pdf; McKinsey (2020) Transforming healthcare with AI: the impact on the workforce and organizations. Available at https://eithealth.eu/wp-content/uploads/2020/03/EIT-Health-and-McKinsey_Transforming-Healthcare-with-AI.pdf; McKinsey (2019) Leading your organization to responsible AI. Available at https://www.mckinsey.com/business-functions/mckinsey-analytics/our-insights/leading-your-organization-to-responsible-ai.

124 McKinsey (2020) Transforming healthcare (..), cit., par. 4.6, pp. 96–101.

125 Available at https://ec.europa.eu/info/publications/white-paper-artificial-intelligence-european-approach-excellence-and-trust_en.

126 All quotes from https://blogs.microsoft.com/on-the-issues/2018/12/06/facial-recognition-its-time-for-action/.

127 All quotes from Christina Montgomery & Ryan Hagemann (2019) “Precision Regulation” and Facial Recognition. Available at https://www.ibm.com/blogs/policy/facial-recognition/.

128 For reasons of space, I do not go into the juridical details of the many laws that are (being) proposed or have passed—but that information is available on request.

129 Note that also in Europe, civil society organizations—like the European Digital Rights (EDRi) association—are targeting new technologies (including facial recognition software) that appear to threaten civil rights and liberties.

130 In the words of Senator Carlyle who sponsored the bill: ‘It’s fair to say Amazon has a deep scepticism and concern about meaningful restrictions on facial recognition’. See https://www.seattletimes.com/business/microsoft-backs-washington-states-facial-recognition-bill-as-amazon-mulls-support/.

131 https://blogs.microsoft.com/on-the-issues/2018/12/06/facial-recognition-its-time-for-action/.

132 Cf. comments in https://blogs.microsoft.com/on-the-issues/2020/03/31/washington-facial-recognition-legislation/.

133 Thomas Metzinger (2019) Ethics washing made in Europe. *Tagesspiegel* 8 April 2019. Available at https://www.tagesspiegel.de/politik/eu-guidelines-ethics-washing-made-in-europe/24195496.html.

134 Dillon Reisman, Jason Schultz, Kate Crawford, & Meredith Whittaker. Algorithmic impact assessments: A practical framework for public agency accountability. New York: AI Now Institute. Available at https://ainowinstitute.org/aiareport2018.pdf.

135 Partnership on AI (2019) Report on Algorithmic Risk Assessment Tools in the U.S. Criminal Justice System. Available at https://www.partnershiponai.org/report-on-machine-learning-in-risk-assessment-tools-in-the-u-s-criminal-justice-system/.

136 Partnership on AI (2019) Report (..), cit., p. 31.

137 Partnership on AI (2019) Report (..), cit., p. 29.
